# Novel Paired Cell Lines for the Study of Lipid Metabolism and Cancer Stemness of Hepatocellular Carcinoma

**DOI:** 10.3389/fcell.2022.821224

**Published:** 2022-05-26

**Authors:** Yun-Hsin Cheng, Ying-Chieh Ko, Hsiang-Ju Ku, Ching-Chun Huang, Yu-Ching Yao, Yi-Tzu Liao, Ying-Tsong Chen, Shiu-Feng Huang, Li-Rung Huang

**Affiliations:** ^1^ Institute of Molecular and Genomic Medicine, National Health Research Institutes, Miaoli, Taiwan; ^2^ Department of Biological Science and Technology, China Medical University, Taichung, Taiwan; ^3^ Institute of Genomics and Bioinformatics, National Chung Hsing University, Taichung, Taiwan; ^4^ Biotechnology Center, National Chung Hsing University, Taichung, Taiwan; ^5^ Graduate Institute of Clinical Medicine, National Taiwan University, Taipei, Taiwan

**Keywords:** hepatocellular carcinoma, cancer stemness, monounsaturated fatty acid (MUFA), myeloid-derived suppressor cells (MDSCs), chemokine

## Abstract

There are few well-characterized syngeneic murine models for hepatocellular carcinoma (HCC), which limits immunological studies and the development of immunotherapies for HCC. We previously established an oncogene-induced spontaneous HCC mouse model based on transposon-mediated oncogene (*AKT* and *NRASV12*) insertion into the genome of hepatocytes to induce tumorigenesis. Two tumor clones with different levels of lipid droplets (LDs) showed similar *in vitro* growth but distinctive *in vivo* phenotypes, including divergent proliferative capability and varying induction of myeloid-derived suppressor cells (MDSCs). The two clones showed distinct gene expression related to lipid metabolism, glycolysis, and cancer stemness. Endogenous fatty acid (FA) synthesis and exogenous monounsaturated fatty acid (MUFA) consumption promoted both tumor proliferation and cancer stemness, and upregulated c-Myc in the HCC cell lines. Moreover, the LD^hi^ HCC cell line expressed a higher level of type II IL-4 receptor, which promoted tumor proliferation through binding IL-4 or IL-13. The chromosomal DNA of two tumor clones, NHRI-8-B4 (LD^hi^) and NHRI-1-E4 (LD^lo^) showed five identical *AKT* insertion sites in chromosomes 9, 10, 13, 16 and 18 and two *NRAS* integration sites in chromosomes 2 and 3. Herein, we describe two novel HCC cell lines with distinct features of lipid metabolism related to cancer stemness and differential interplay with the immune system, and present this syngeneic HCC mouse model as a practical tool for the study of cancer stemness and discovery of new therapies targeting liver cancers.

## Introduction

Liver cancer is the seventh most common cancer worldwide, and causes approximately 780,000 deaths annually. Liver cancers comprise primarily hepatocellular carcinoma (HCC; 85%–90%) and cholangiocarcinoma. Chronic HBV and HCV infection, alcohol hepatitis, and nonalcoholic fatty liver diseases (NAFLD) are three major etiologies of HCC and all cause chronic inflammation or steatohepatitis before HCC progression (Llovet et al., 2021). Overnutrition, which may result in overweight or obesity, is the primary cause of NAFLD. The most severe form of NAFLD is nonalcoholic steatohepatitis (NASH). There is strong link between body fat and cancers (Lauby-Secretan et al., 2016). Obesity and overweight are associated with increased incidence of a several cancers, e.g., liver, colorectal, breast, pancreatic and kidney cancers. Increased fatty acid (FA) uptake by a number of tumor cell types has been observed in high-fat-diet induced obese tumor-bearing mice and local deprivation of free fatty acids (FFA) by tumor cells in the tumor microenvironment (TME) jeopardized CD8^+^ T cell responses (Ringel et al., 2020). Upregulation of *de novo* FA synthesis and aberrant fatty acid oxidation (FAO), which contribute to tumor initiation and progression, are observed in various types of cancers, including HCC (Medes et al., 1953; Kuhajda et al., 1994; Kuhajda, 2000; Budhu et al., 2013). Fatty acid synthase (FASN) is identified as one of the key lipogenic enzymes regulating *de novo* FA synthesis in cancer cells (Menendez and Lupu, 2007). Expression or activation of sterol regulatory element-binding protein 1 (SREBP1), which transcriptionally activates several lipogenic enzymes, including FASN, is modified by phosphatidylinositol-3 kinase (PI3K)–Akt and extracellular signal-regulated kinases (ERK1 and ERK2) active in hormone-sensitive tissues, lipogenic tissues, or tumors (Menendez and Lupu, 2007). Activation of the Akt signaling prevents post-transcriptional degradation of *FASN*, *SREBP1* and *SREBP2* and therefore enhances *de novo* FA synthesis, which facilitates HCC development (Calvisi et al., 2011).

Experimental animal models are used to recapitulate the genetic alteration, metabolic stress, and immunological signaling cascade induced during HCC initiation and progression, and are essential in understanding the immunopathogenesis of HCC and for development of targeted therapeutics. Genetically engineered, chemically induced allograft and xenograft HCC mouse models are commonly used tools for studies of pathogenesis and efficacy of anti-cancer drugs and immunotherapies. The unique anatomy of the liver allows hydrodynamic injection (HDI) of naked DNA plasmids into the nuclei of hepatocytes (Liu et al., 1999), which allow integration of the gene of interest into chromosomes in the context of transposon-mediated transposition. The sleeping beauty (SB) transposon system of carrying oncogenes is now widely used to establish HCC mouse models (Chen and Calvisi, 2014).

We previously established an Akt/N-Ras-induced HCC mouse model in which the tumor expressed luciferase and surrogate tumor-associated antigens (TAA). Tumor growth can be monitored using an *in vivo* imaging system (IVIS) and TAA-specific T cells are induced but exhausted in the TME (Liu et al., 2018). The Akt and ERK signaling pathways were both activated in Akt/N-Ras-induced liver cancers (Ho et al., 2012) and aberrant lipid metabolism was observed in the originally transformed hepatocytes, as well as in the tumor cells (Liu et al., 2018). To further characterize Akt/N-Ras-induced liver cancers, we isolated tumor cell clones from the tumor mass for analysis. Although both the Akt and NRAS pathways were activated in the isolated tumor clones, we observed clone-specific differences in lipid content, cancer stemness, *in vivo* growth, and interplay with immune cells. The clone with high lipid content upregulated gene sets for lipid uptake, FA synthesis, cancer stemness, and chemokine and cytokine receptors. The exogeneous FA consumption and *de novo* FA synthesis contributed to the cancer stemness of the isolated HCC clones, whereas the cytokine receptor signaling, including receptors for IL-4 and IL-13, enhanced tumor growth. This study clearly demonstrates that delivery of transposons carrying oncogenes via HDI induces heterogenous cancer cells in the liver even though the transposition of SB transposon targets several identical TA-rich regions of the chromosomes. Ultimately, we established and characterized HCC cell lines that may ease the shortage of reliable syngeneic HCC mouse models for use in immunological studies and drug discovery.

## Materials and Methods

### Animal Studies and HCC Induction by HDI

Male C57BL/6j mice at 4–5 weeks old were purchased from the National Laboratory Animal Center (Taipei, Taiwan) and kept in the laboratory animal center (LAC) of NHRI. The mice received 2 μg of pCMV(CAT)T7-SB100 (Addgene #34879), 10 μg of pT/Caggs-NRASV12 (Addgene #20205), and 10 μg of pKT2/CLP-AKT-LUC plasmids through HDI and were monitored for tumor growth weekly using IVIS. Detailed procedures were described in our previous study (Liu et al., 2018).

### HCC Clones and Cell Culture

The liver of each HCC-bearing mouse was perfused using prewarmed Gey’s balanced salt solution (GBSS) followed by DMEM-based medium with 0.5 mg/ml collagenase IV. After perfusion, the liver was collected and cut to release cells into medium. The cell suspension was cultured until colonies formed. Colonies were transferred to new culture wells for continuous growth. Single cells that were platelet-derived growth factor receptor alpha-negative (PDGFRα^−^) CD45^−^ CD44^+^ were sorted from each colony for continued culture. The resultant HCC clones were further characterized.

A total of 5 × 10^3^ HCC cells were added to 100 μl DMEM-based medium and incubated for 24, 48, or 72 h. Cell number was assessed by adding 150 μg/ml luciferin and measuring luminescence *via* Hidex at indicated time points.

For sorafenib, fatostatin, TVB-2640, CAY10566, and triclosan treatments, 1 × 10^3^ or 5 × 10^3^ HCC cells were seeded and then treated with titrated concentrations of each drug. Luciferase activity/cell number was measured at 72 h after treatment.

For FA treatment, 1 × 10^3^ HCC cells were cultured in RPMI-based medium supplemented with 1% defatted bovine serum albumin (BSA) and then treated with 150 μM of the indicated FAs for 72 h.

### Sphere Formation and Quantification Assay

A total of 2.5 × 10^3^ HCC cells in 10 μl medium were mixed 1:1 with Matrigel and used to seed a 24-well plate. Cells were incubated at 37°C for 1 h before adding an additional 500 μl medium. For general sphere formation assays, serum-free DMEM-based medium was used and refreshed every 2–3 days. Sphere formation was observed and digital images were captured at days 4, 7, and 10 using a Leica AF6000 microscope. For inhibitor treatment, DMEM-based medium with 1% FBS plus inhibitors, at indicated concentrations, were used for cell culture in non-coating tissue culture plates; the medium with inhibitors was refreshed every 2–3 days until day 7. For FA treatment, RPMI-based medium with 1% defatted BSA plus 150 μM of the indicated FAs was used and refreshed every 2–3 days until day 7. The number and diameter of spheres were quantified using LAS X (Leica microsystem software). Spheres with a diameter of more than 40 μm were counted. Sphere formation efficiency (SFE) was calculated using the formula: (number of spheres counted ÷ number of cells seeded cells) × 100.

### Nanopore Whole Genome Shotgun Sequencing

Genomic DNA purification from cultured cell lines was performed using the DNeasy Blood and Tissue Kit (QIAGEN). Sequencing libraries were prepared using the Rapid Sequencing Kit (Oxford Nanopore Technologies) and sequencing was performed using R9.4 Flow Cells on a MinION Mk1C sequencer (Oxford Nanopore Technologies) following standard protocols. Basecalled reads passing sequence quality control were analyzed for the presence of the marker sequences NRAS (Addgene #20205) or AKT-LUC (Liu et al., 2018) using blastn (NCBI). In summary, 1.68 M reads (>8.7 Gb total nucleotides) obtained from NHRI-8-B4, 2.06 M reads (>12 Gb total nucleotides) obtained from NHRI-1-E4, 1.15 M reads (>5.1 Gb total nucleotides) obtained from NHRI-1F-1, 1.02 M reads (>6.37 Gb total nucleotides) obtained from NHRI-8-A4, and 1.24 M reads (>6.71 Gb total nucleotides) obtained from NHRI-8-C4 were analyzed. The flanking sequences of the markers were mapped to the Mus musculus C57BL/6 J genome assembly (ASM377452v2) using blastn to identify the integration sites of the markers. Annotation of the genomic regions containing the integration was performed using blastn and the Mus musculus genome (GRCm39 reference Annotation Release 109) from NCBI as reference.

### RNA-Seq Analysis

Total RNA from cultured cells was purified using the RNeasy mini kit (QIAGEN) and RNA quality was checked using a Qubit Fluorometer (Thermo Fisher Scientific). RNA-Seq analyses were performed using an illumina NovaSeq 6,000 system in 6G depth in the DNA Sequencing Core Laboratory of NHRI. Genes involved in lipid metabolism, glycolysis, cancer stemness, as well as those for cytokine receptors and chemokines in NHRI-1-E4, NHRI-1-F1, NHRI-8-A4, NHRI-8-B4, NHRI-8-C4, #1 (pool) and #8 (pool) cells were shown in the heatmap (*via* Heatmapper). Gene set enrichment analyses (GSEA) were performed using GSEA 4.1.0 software. PCA plot was performed using R prcomp.

### Real-Time Quantitative Polymerase Chain Reaction

Total RNA from cultured cells or homogenized tumor tissues was purified using TRIzol reagent (Thermo Fisher Scientific) and reverse transcribed into cDNA using EvoScript Universal cDNA Master (Roche). The KAPA SYBR FAST qPCR Kit (Kapa Biosystems) and corresponding primer sets (Supplementary Table S1) were used for quantitative real-time PCR analysis via the Bio-Rad CFX96 Real-time System. Relative expression was calculated using the 2^−ΔCt^ method with normalization to *β*-actin.

### Induction of MDSCs *in vitro*

Bone marrow cells at 1 × 10^6^ cells per ml in RPMI-based medium or HCC conditional medium were treated with or without GM-CSF (20 ng/ml) for 5 days. After a 3-day induction, the culture medium was refreshed, and the cells were cultured for another 2 days before analysis. HCC conditional medium was harvested from supernatant of 24 h-HCC cell culture in RPMI-based medium.

### Statistical Analysis

GraphPad Prism 8 (GraphPad Software) was used for statistical analysis. Unpaired student’s t-test was used for comparisons of two groups, one-way ANOVA was used for comparisons of multiple groups, and two-way ANOVA was used for multiple comparisons, such as tumor growth curves and marker expression. Data represent mean ± SD of indicated numbers of biological repeats. Statistical significance is represented *via p* values in figures, and data are labeled as not detected (nd); not significant (ns); *p* > 0.05; *p < 0.05; **p < 0.01; ***p < 0.001; or ****p < 0.0001.

Detailed methods were included in our previous reports (Jian et al., 2017; Liu et al., 2018) and/or can be found in Supplementary Material.

## Results

### Hepatocellular Carcinoma Cell Lines from an *AKT* and *NRAS*-Driven HCC Mouse Possess Features Distinct from Hepa1-6 Cells

**FIGURE 1:**
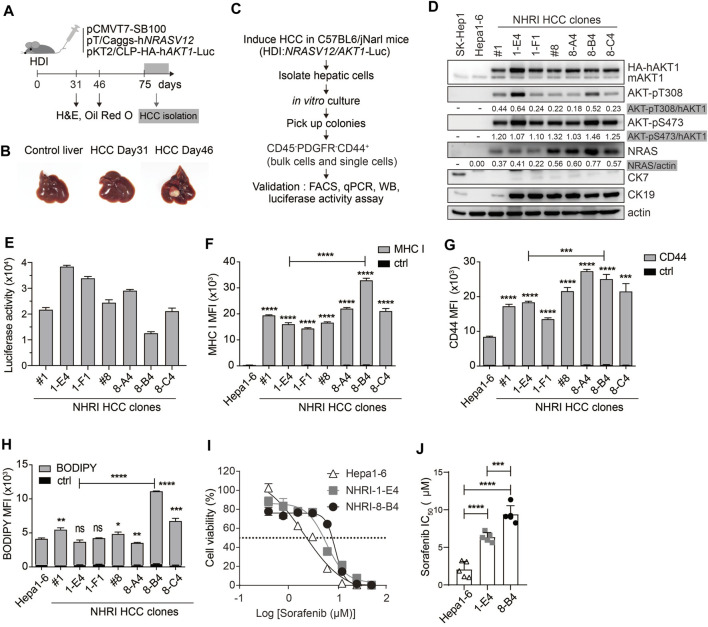
Isolation and characterization of HCC cell lines from Akt1/N-Ras-induced HCC mice. **(A)** Schematic representation of hydrodynamic injection (HDI)-induced hepatocellular carcinoma (HCC) mouse model. HCC-bearing livers were dissected at indicated time points. **(B)** Images of control (normal) and HCC-bearing livers after dissection. **(C)** Flow chart describing HCC clone isolation and characterization. **(D)** Expression of indicated proteins in Hepa1-6 cells, SK-Hep1 and isolated HCC clones. **(E)** Luminescence of HCC clones (1 × 10^4^). *n* = 4. **(F)** Mean fluorescence intensity (MFI) of MHC class I expression, **(G)** MFI of CD44 expression, and **(H)** neutral lipid content detected by BODIPY 493/503 in HCC clones compared to Hepa1-6 cells or to each other as indicated. *n* = 3. **(I)** Cytotoxicity (%) of Hepa1-6, NHRI-1-E4, and NHRI-8-B4 cells with sorafenib treatment for 72 h. *n* = 4. **(J)** Sorafenib half maximal inhibitory concentration (IC_50_; μM) of Hepa1-6, NHRI-1-E4, and NHRI-8-B4 cells. *n* = 5. Data are representative of two **(E–H)** or five **(I)** independent experiments. ns, not significant; *p < 0.05; **p < 0.01; ***p < 0.001; ****p < 0.0001 *via* unpaired Student’s *t*-test **(F–H)** or one-way ANOVA **(I)**.

Activation of AKT-mTOR and RAS-MAPK signaling pathways are observed in many cancer types, including HCC (Llovet et al., 2021). Our lab has established a spontaneous HCC mouse model through HDI of three plasmids encoding SB transposase, human NRAS (G12V), and human AKT1 and luciferase (Liu et al., 2018). Tumor lesion was not yet observed at day 31 but already showed in the liver at day 46 after induction (Figures 1A,B). Massive lipid accumulation in the liver and the tumor was observed after HDI, resulting from activation of Akt signaling (Supplementary Figure S1A).

We isolated HCC clones from this transposon-based HCC mouse model for phenotypic analysis. We collected hepatic cells from tumor masses from C57BL/6 J mice at day 75 following HDI. Cells were cultured *in vitro* for 8 days, then colonies were selected from the cultures to further isolate cells with the CD45^−^ PDGFRα^−^ CD44^+^ profile, thereby excluding cells of hematopoietic origin and fibroblasts through single cell FACSorting (Figure 1C). The HCC clones grown from the sorted pooled colonies (#1, #8) and sorted single cells (1-E4, 1-F1, 8-A4, 8-B4, and 8-C4) were subjected to further characterization and comparison with the widely used murine HCC cell line, Hepa1-6 cells (Kröger et al., 2001). Hepa1-6 cells can grow in immunocompetent mice and serve as a syngeneic mouse model; however, Hepa1-6 cells express low levels of MHC class I proteins. Compared to Hepa1-6 cells, our novel tumor cell lines overexpressed activated AKT and NRAS, but there were no differences in Akt expression and significant differences in NRAS expression among these Akt1/N-Ras-induced tumor cell lines (Figure 1D; Supplementary Figure S1B).

All the Akt1/N-Ras-induced tumor cell lines and Hepa1-6 expressed nearly no CK7/*Krt7* (keratin 7) (Figure 1D; Supplementary Figure S1C), and the Akt1/N-Ras-induced tumor cell lines but not Hepa1-6 expressed significant amount of the biliary/progenitor cell marker, CK19/*Krt19* (keratin 19) (Figure 1D; Supplementary Figure S1D), which suggests that these Akt1/N-Ras-induced tumor cell lines possess the properties of hepatic progenitor cells but are not derived from cholangiocytes. To confirm tissue specificity, we found that the HCC clones expressed liver-specific *Alb* (albumin) although at a lower level compared to Hepa1-6 cells (Supplementary Figure S1E). The HCC clones expressed higher mRNA levels of HCC markers *Krt18* (keratin 18, encodes CK18) and *Fuca1* (alpha-L-fucosidase, encodes AFU) (Wells et al., 1997; Wang et al., 2014) than Hepa1-6 cells (Supplementary Figures S1F,G). However, the HCC clones expressed nearly no *Afp* (Alpha-Fetoprotein) nor *Gpc3* (glypican 3), two well-known HCC markers, which could be detected in Hepa1-6 cells (supplementary Figures S1H,I). Due to *AKT1*/luciferase gene transposition, the HCC clones exhibited bioluminescent reporter gene activity (Figure 1E), as well as higher levels of MHC class I protein (H-2K^b^, Figure 1F) and the cancer stem cell (CSC) marker CD44 (Figure 1G) compared to Hepa1-6 cells. The expression of MHC class I protein in HCC clones can be an advantage in cancer immunotherapy research.

### NHRI-8-B4 Cells Exhibit Stronger Sorafenib Resistance than NHRI-1-E4 and Hepa1-6 Cells

We observed lipid storage in the liver and tumor tissues in Akt/N-Ras-induced HCC, and we therefore measured the neutral lipid content using BODIPY 493/503 staining followed by FACS analysis. These HCC clones contained lipid content similar to Hepa1-6, with the exception of NHRI-8-B4 and NHRI-8-C4, which had a higher LD level than the other cell lines (Figure 1H). We then chose the NHRI-1-E4 and NHRI-8-B4 cell lines for further characterization and to study the influence of LD on phenotypes of liver cancer cells.

Sorafenib, a multiple-target tyrosine kinase inhibitor, is a first-line therapy for HCC. However, most patients do not benefit from treatment solely with sorafenib. Previous studies have shown that activation of Akt and N-Ras signaling pathways contribute to sorafenib resistance (Chen et al., 2011; Dietrich et al., 2019). Thus, we examined whether NHRI-8-B4 or NHRI-1-E4 cells were more resistant to sorafenib than Hepa1-6 cells. HCC cells were cultured in the presence of serially titrated sorafenib for 72 h. Sorafenib-induced cytotoxicity was measured using the *in vitro* luciferase activity assay. NHRI-1-E4 and NHRI-8-B4 cells were more resistant to sorafenib than Hepa1-6 cells (Figure 1I). The half maximal inhibitory concentrations (IC_50_) of Hepa1-6, NHRI-1-E4, and NHRI-8-B4 cells were 2.1 ± 1.1 μM, 6.4 ± 0.6 μM, and 9.4 ± 1.2 μM, respectively (Figure 1J). NHRI-8-B4 exhibited stronger resistance toward sorafenib than NHRI-1-E4 cells may due to its higher expression level of NRAS (Dietrich et al., 2019).

### Analyses of Sites of *AKT* or *NRAS* Cassette Integration in NHRI HCC Clones

**FIGURE 2:**
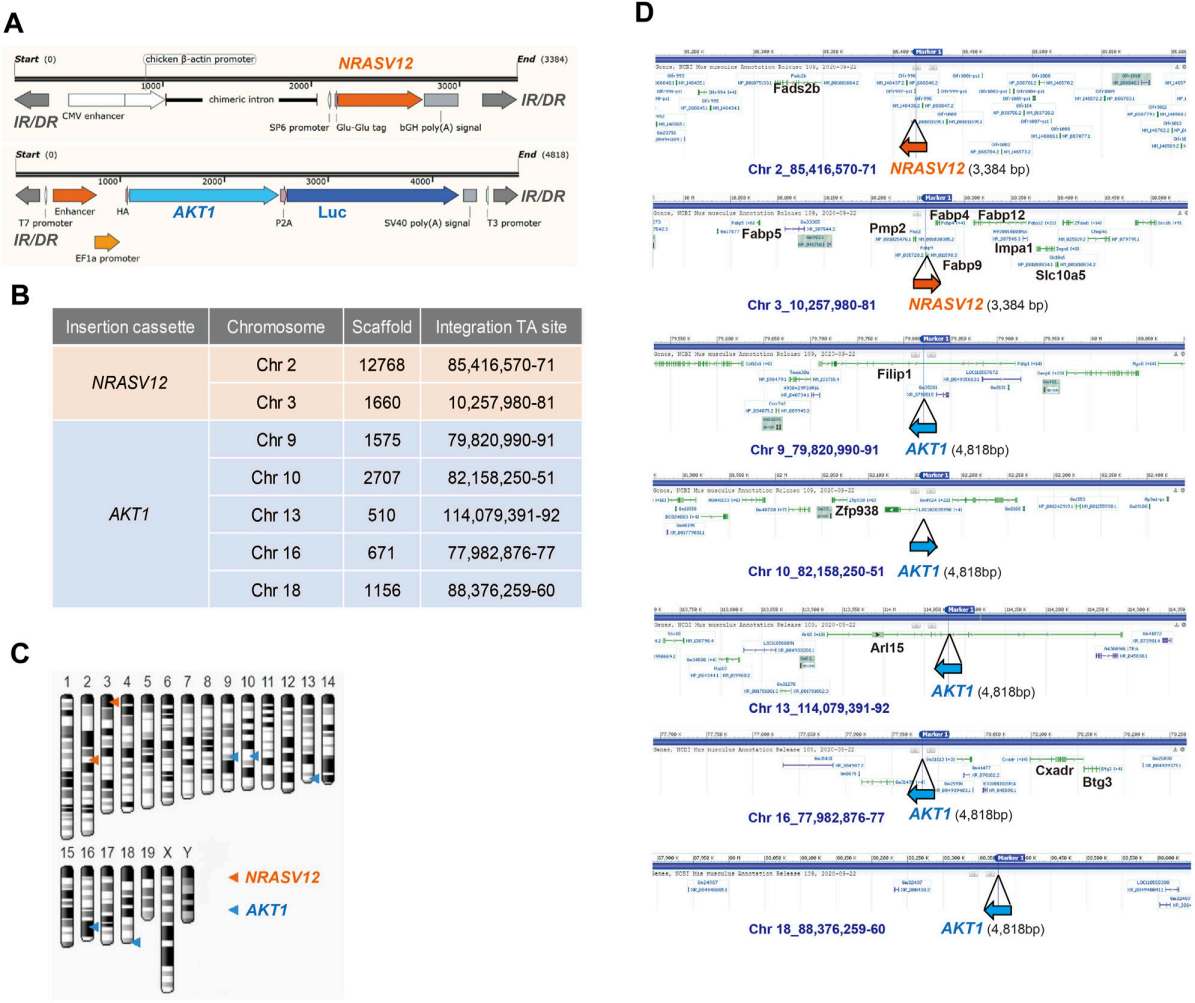
Whole-genome sequencing of NHRI HCC cell lines with Nanopore technology. **(A)** Schematic representation of *NRAS* and *AKT* integration cassettes. Luc: luciferase; IR/DR: inverted repeats and direct repeats. **(B)** Insertion sites of *NRAS* and *AKT* cassettes in all NHRI HCC clones determined by Nanopore technology. **(C)** Schematic representation of *NRAS* and *AKT* insertions in chromosomes of NHRI HCC clones. **(D)** Genes neighboring each insertion site.

Human *NRASV12* (*NRAS* G12V), and human *AKT1* plus the luciferase gene, flanked by terminal inverted repeats and short direct repeats (IR/DR) in the two transposons (Figure 2A) can integrate into chromosomes randomly through the cut and paste mechanism of SB transposase (Izsvák and Ivics, 2004). We performed whole genome shotgun sequencing using the Nanopore system to reveal the integration sites of *AKT1* and *NRAS* cassettes in chromosomes of NHRI HCC clones. The sequencing coverage was 4.41x, 1.9x, 2.4x, 3.2x, and 2.5x for the genomic DNA samples from 1-E4, 1-F1, 8-A4, 8-B4, and 8-C4, respectively. Surprisingly, we detected and mapped two identical *NRAS* insertions in chromosome 2 and 3 and five identical *AKT1* insertions, one each in chromosomes 9, 10, 13, 16, and 18, in each HCC clones (Figures 2B–D). The integration sites in each HCC clones were further confirmed by targeted PCR sequencing with primers designed for the 5' and 3' junctions of each integration site (Supplementary Figures S2A,B; Supplementary Table S4). The genomic DNA from Hepa1-6 was served as negative control for the targeted PCR. The sequence alignment results demonstrated that all identified insertion sites were TA sites. The SB transposase-mediated transposition led to a duplicated TA site and resulted in two TA sites flanking the *AKT* or *NRAS* cassettes (Supplementary Figure S2B). Herein, we excluded the possibility that the differential features such as lipid content or drug resistance resulted from different integration sites of *AKT1* or *NRASV12* in the genome of each HCC clone. The differential features in the HCC clones with the same *AKT1*/*NRAS* integration sites may be due to the intratumoral differentiation and *in vitro* selection of the HCC clones originated from the same transformed hepatocyte which gained growth advantage in the very beginning of tumor progression.

### Transcriptional Profiles of NHRI-8-B4 Are Similar to the *CTNNB1* subclass of Human HCC with Aberrant Lipid Metabolism

**FIGURE 3:**
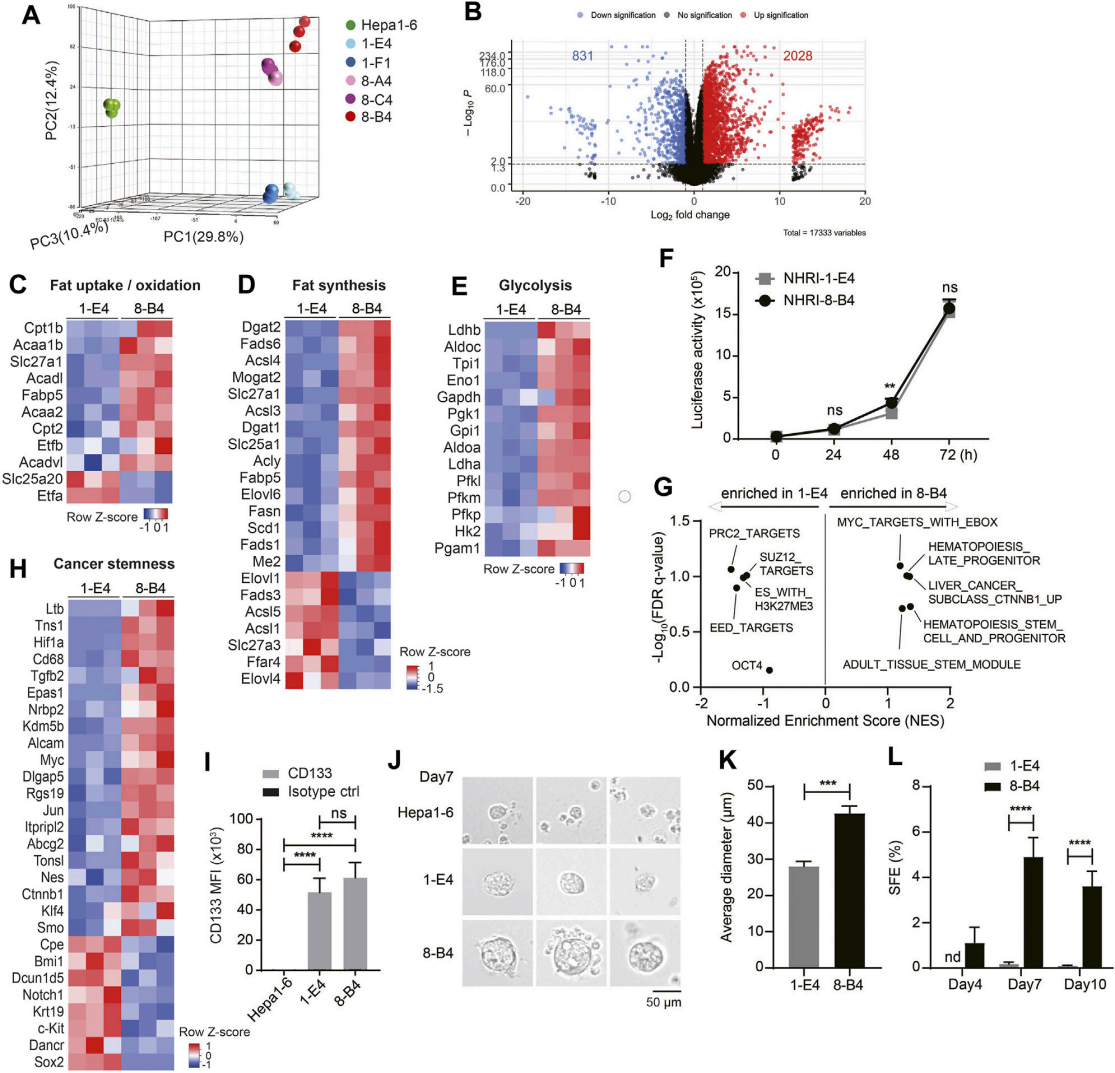
Transcriptional analyses show higher lipid metabolism and stemness in NHRI-8-B4 than NHRI-1-E4 cells. **(A)** Three-dimensional principal component analysis (PCA) visualizing the RNA-seq results of indicated HCC cell lines. **(B)** Volcano plot of RNA-Seq results showing log2 fold change versus -log *p* value. Heatmaps of genes involved in fat uptake/oxidation **(C)**, fat synthesis **(D)** and glycolysis **(E)**in NHRI-1-E4 and NHRI-8-B4 cells. **(F)** *In vitro* growth curves were generated based on luciferase activity. *n* = 6. **(G)** GSEA of cancer stemness gene sets. Normalized enrichment score (NES) > 0 indicates gene sets correlated with NHRI-8-B4 cells rather than NHRI-1-E4 cells. **(H)** Heatmaps of genes involved in cancer stemness. **(I)** CD133 expression on indicated cells. *n* = 3. **(J)** Representative images of spheres formed from indicated cells in serum-free culture for 7 days. Scale bar, 50 μm. **(K)** Average diameter (μm) of spheres measured at day 7. *n* = 3. **(L)** Spheres (diameter >40 μm) from each cell line are quantified by Leica X software to calculate sphere formation efficiency (SFE; %). *n* = 3. Data are representative of three independent experiments **(I–L)**. nd, not detected; ns, not significant; **p < 0.01; ***p < 0.001; ****p < 0.0001 *via* two-way ANOVA **(F, I, L)** or unpaired *t*-test **(K)**.

We further analyzed the transcriptional profiles of each HCC cell line including Hepa1-6 by RNA-Seq. Principal component analysis (PCA) demonstrated that NHRI HCC clones had profiles distinct from Hepa1-6 (Figure 3A). The transcriptional profiles of NHRI HCC clones were clustered based on their origin. NHRI-1-E4 and NHRI-1-F1 originated from pool #1 had similar transcriptional profiles, whereas NHRI-8-A4, NHRI-8-B4 and NHRI-8-C4 originated from pool #8 were similar to each other at transcriptional level (Figure 3A). NHRI-8-A4, NHRI-8-B4 and NHRI-8-C4 originated from pool #8 showed a distinct gene expression pattern in lipid metabolism and glycolysis in comparison to Hepa1-6 and clones derived from pool #1 (Supplementary Figure S3). We therefore chose the NHRI-1-E4 and NHRI-8-B4 cell lines for further characterization. There were 2028 genes significantly upregulated and 831 genes downregulated in NHRI-8-B4 cells compared to NHRI-1-E4 (Figure 3B). Heatmaps showed that genes involved in fat uptake/oxidation, fat synthesis, and glycolysis were upregulated in NHRI-8-B4 cells compared to NHRI-1-E4 cells (Figures 3C–E), which suggests that NHRI-8-B4 cells may utilize lipid and glucose metabolism to sustain their high proliferative capability per the Warburg effect. Elevated expression of genes involved in lipid uptake, synthesis, elongation, desaturation and storage in NHRI-8-B4 cells was confirmed by quantitative PCR (Supplementary Figures S4A–D), which may explain their higher lipid content compared to NHRI-1-E4 (Figure 1H, Supplementary Figure S4E). However, the higher utilization of lipid and glucose metabolism in NHRI-8-B4 cells did not result in a faster growth rate compared to NHRI-1-E4 cells, as revealed by *in vitro* luciferase activity (Figure 3F).

GSEA revealed that NHRI-8-B4 cells expressed distinct sets of genes related to cancer stemness, including CHIANG LIVER CANCER SUBCLASS CTNNB1 UP (Chiang et al., 2008) (normalized enrichment score (NES) = 1.36, false discovery rate (FDR) *q* value = 0.10), IVANOVA HEMATOPOIESIS STEM CELL AND PROGENITOR (Ivanova et al., 2002) (NES = 1.36, FDR *q* value = 0.19), and IVANOVA HEMATOPOIESIS LATE PROGENITOR (Ivanova et al., 2002) (NES = 1.31, FDR *q* value = 0.10), whereas NHRI-1-E4 cells expressed other stemness related gene sets, including BENPORATH PRC2 TARGETS (NES = −1.51, FDR *q* value = 0.09), BENPORATH EED TARGETS (NES = −1.42, FDR *q* value = 0.13), and BENPORATH ES WITH H3K27ME3 (NES = -1.31, FDR *q* value = 0.10) (Figure 3G) (Ben-Porath et al., 2008). Expression of the *Ctnnb1*, *Myc* (downstream of the WNT/*β*-catenin signaling pathway), *Hif1α*, *Tgfb2*, *Alcam*, and *Ltb* genes were enriched in NHRI-8-B4 cells (Figure 3H, Supplementary Figure S4F). HIF and Myc crosstalk has been proposed, and the overexpression of Myc has been shown to increase HIF expression and activity (Li et al., 2020). The distinct patterns of expression of genes involved in cancer stemness between NHRI-1-E4 and NHRI-8-B4 did not affect their *in vitro* proliferative capacity, but have the potential to affect their cancer stemness and *in vivo* cancer progression.

### NHRI-8-B4 Cells Show Stronger Cancer Stemness than NHRI-1-E4 Cells

Both the NHRI-1-E4 and NHRI-8-B4 cell lines were CD44^high^ and CD133^high^, whereas Hepa1-6 cells expressed low levels of CD44 and CD133 (Figures 1G, 3I). Consistent with transcriptional profiles, both NHRI-1-E4 and NHRI-8-B4 were cancer stem cell-like cell lines. We further examined whether there was variation in cancer stemness function by sphere formation assay (Bahmad et al., 2018). Equal numbers of the three HCC cell lines, Hepa1-6, NHRI-1-E4, and NHRI-8-B4 cells, were seeded (equal cell number confirmed in Supplementary Figure S4G) and sphere formation was observed at days 4, 7, and 10. Significant sizes and numbers of spheres were observed in NHRI-1-E4 and NHRI-8-B4 cultures starting at day 7, whereas fewer and smaller spheres were observed in Hepa1-6 culture (Figure 3J). The average diameter of spheres formed from NHRI-1-E4 cells (28.03 ± 1.38 μm) were much smaller than those from NHRI-8-B4 cells (42.62 ± 2.03 μm) at day 7 (Figures 3J,K). The sphere formation efficiency (SFE) of NHRI-8-B4 cells peaked at day 7 at 4.9%, whereas the SFE of NHRI-1-E4 cells was 0.17% at day 7 (Figure 3L), which suggests that NHRI-8-B4 cells are higher in stemness compared to NHRI-1-E4 cells.

### Endogenous and Exogenous Lipids Are Required for Sphere Formation

**FIGURE 4:**
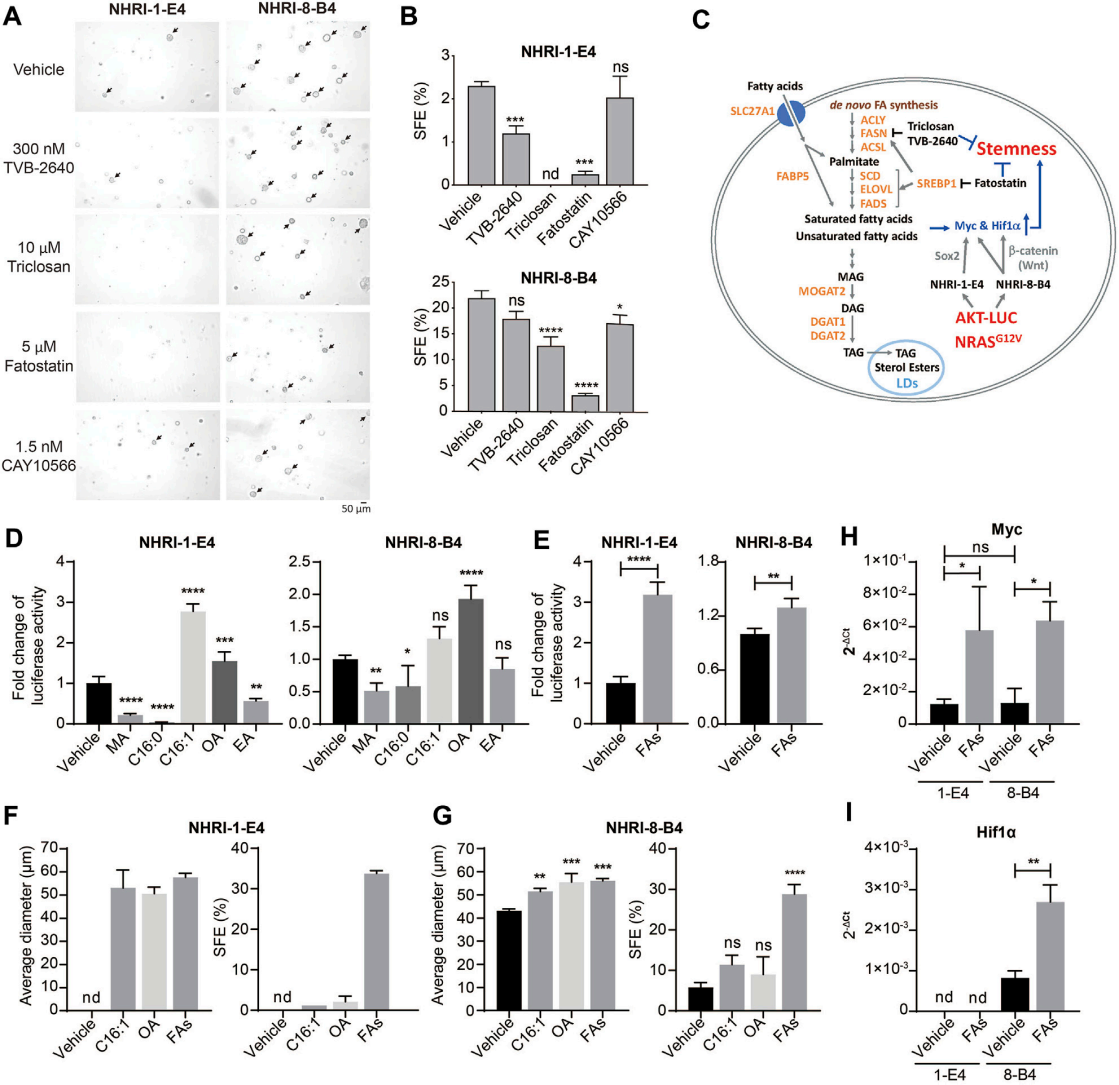
Endogenous and exogenous lipids contribute to cell proliferation and stemness of NHRI-1-E4 and NHRI-8-B4 cells. **(A)** Representative spheres, indicated by arrows, formed at day 7 in the presence of indicated inhibitors. **(B)** SFE (%) from **(A)**. *n* = 3. **(C)** Schematic representation of crosstalk between lipid metabolism and cancer stemness in NHRI-1-E4 and NHRI-8-B4 cells. Growth of NHRI-1-E4 and NHRI-8-B4 for 72 h with 150 μM indicated fatty acid **(D)** or 150 μM multiple fatty acids (FAs) **(E)**; combination of five fatty acids used in **(D)**. *n* = 4. **(F,G)** Average diameter (μm) of spheres and SFE (%) of each cell line with indicated treatment for 7 days. *n* = 3. Levels of Myc **(H)** and Hif1α **(I)** mRNA in NHRI-1-E4 and NHRI-8-B4 cells with 150 μM multiple fatty acid treatment (FAs) for 24 h. *n* = 3. Data are representative of two **(A,B,F,G)**, four **(D,E)**, or three **(H,I)** independent experiments. nd, not detected; ns, not significant; *p < 0.05; **p < 0.01; ***p < 0.001; ****p < 0.0001 via one-way **(B,D,G)** or two-way **(H)** ANOVA, or unpaired *t*-test **(E,I)**. MA, myristic acid; C16:0: palmitic acid; C16:1: palmitoleic acid; OA, oleic acid; EA, erucic acid.

Lipid metabolism, including *de novo* FA synthesis and lipid desaturation, are involved in cancer pathogenesis in many cancer types (Menendez and Lupu, 2007). We therefore examined whether activated lipid metabolism in NHRI-8-B4 cells contributed to their high degree of cancer stemness. *De novo* lipid synthesis can be inhibited by the FASN inhibitors triclosan and TVB-2640 and the SREBP inhibitor fatostatin. NHRI-1-E4 and NHRI-8-B4 cells were able to tolerate triclosan, TVB-2640 and fatostatin at concentrations of 20 μM, 300 nM and 5 μM, respectively, during *in vitro* proliferation (Supplementary Figures S5A,B). We then treated the HCC cells with 10 μM triclosan, 300 nM TVB-2640 and 5 μM fatostatin, respectively, in the sphere formation assay. Inhibition of FASN by triclosan or TVB-2640 caused significant reduction of sphere formation in NHRI-1-E4 cells, but only slight reduction in NHRI-8-B4 cells (Figure 4A, arrows). This suggests that NHRI-1-E4 cells, but not NHRI-8-B4 cells, rely on *de novo* FA synthesis to maintain their cancer stemness. SREBP1 is a master transcriptional factor for lipid metabolism and is activated by PI3K/Akt signaling, which subsequently regulates not only expression of genes involved in *de novo* lipogenesis (FASN, ACC), but also genes involved in FA re-esterification, FA desaturation, and elongation (Hagen et al., 2010; Guo et al., 2014). Therefore, fatostatin treatment dramatically reduced sphere formation in both NHRI-1-E4 and NHRI-8-B4 cells (Figures 4A,B), suggesting increased FA re-esterification, FA desaturation, or elongation by activation of SREBP1 may play an important role in cancer stemness (Figure 4C). We then treated the two cell lines with 1.5 nM stearoyl-CoA desaturase1 (SCD1) inhibitor, CAY10566, and found that only the SFE of NHRI-8-B4 but not that of NHRI-1-E4 slightly decreased when FA desaturation was inhibited (Figures 4A,B).

We next examined the role of FAs generated downstream of SREBP1 signaling in the promotion of proliferation and cancer stemness in these HCC cell lines. We observed an increase of neutral lipid content in both NHRI-1-E4 and NHRI-8-B4 when treated with indicated FAs (Supplementary Figures S5C,D). Treatment of saturated FAs, myristic acid (MA, C14:0) and palmitic acid (C16:0), suppressed growth of NHRI-1-E4 and NHRI-8-B4 cells. In contrast, MUFAs, palmitoleic acid (C16:1 n-7) and oleic acid (OA, C18:1 n-9), promoted growth of NHRI-1-E4 and NHRI-8-B4 cells (Figure 4D). NHRI-1-E4 cells favored palmitoleic acid over oleic acid, with the reverse occurring in NHRI-8-B4 cells. Combination of the five examined FAs (MA, palmitic acid, palmitoleic acid, OA, and erucic acid (EA, C22:1n-9) in equal concentration also promoted growth of both NHRI-1-E4 and NHRI-8-B4 cells (Figure 4E). Treatment with a single MUFA (palmitoleic acid or oleic acid) or multiple FAs increased the size and number of spheres formed from either NHRI-1-E4 or NHRI-8-B4 cells (Figures 4F,G). Treatment with the combined five FAs resulted in the most dramatic enhancement of cancer stemness for both NHRI-1-E4 and NHRI-8-B4 cell lines. Thus, we analyzed mRNA levels of stemness related genes and found that expression of *Myc* was upregulated by FA treatment in both cell lines (Figure 4H), whereas upregulated expression of Hif1α by FA treatment only was observed in NHRI-8-B4 (Figure 4I). Together, these results demonstrate that NHRI-1-E4 cells are more dependent on exogenous FAs than NHRI-8-B4 cells for promotion of proliferation and cancer stemness, whereas NHRI-8-B4 cells had a higher lipid metabolic rate and accumulated more endogenous FAs than NHRI-1-E4 cells and therefore less dependent on *de novo* FA synthesis or exogenous FAs.

### NHRI-8-B4 Cells Grow Aggressively and Stably than NHRI-1-E4 Cells or Hepa1-6 *in Vivo*

**FIGURE 5:**
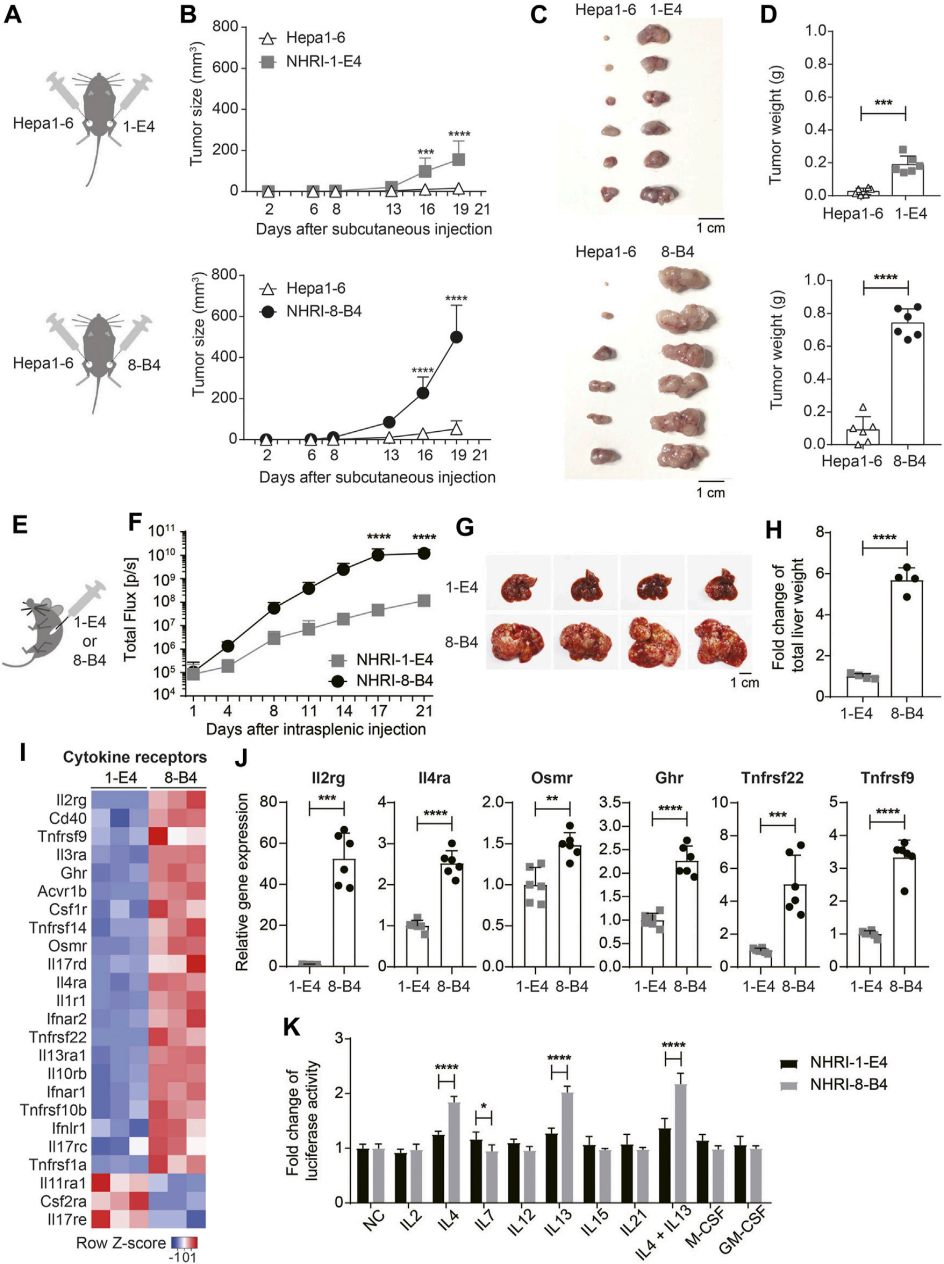
NHRI-8-B4 cells grow more aggressively and stably than NHRI-1-E4 cells or Hepa1-6 *in vivo*. **(A)** Schematic depiction of subcutaneous inoculation of Hepa1-6 (6 × 10^5^) and NHRI-1-E4 (6 × 10^5^) or NHRI-8-B4 (6 × 10^5^) cells in groins of male C57BL/6j mice. **(B)** Subcutaneous tumor size was measured from day 2 to day 19 after inoculation. *n* = 6. Gross appearance **(C)** and tumor weight **(D)** at day 22. *n* = 6. **(E)** Schematic depiction of intrasplenic inoculation of mice with NHRI-1-E4 (1 × 10^5^) or NHRI-8-B4 (1 × 10^5^) cells. **(F)** Hepatic tumor progression. *n* = 4. Liver appearance **(G)** and relative weight **(H)** at day 22. *n* = 4. **(I)** Heatmap of cytokine receptors from RNA-Seq results. **(J)** Levels of mRNA of indicated genes in NHRI-1-E4 and NHRI-8-B4 cells. *n* = 6. **(K)** Cell growth after treatment for 72 h with indicated cytokines at the following concentrations: 250 U/ml IL2, 50 ng/ml IL4, 50 ng/ml IL7, 50 ng/ml IL12, 50 ng/ml IL13, 50 ng/ml IL15, 50 ng/ml IL21, 50 ng/ml IL4 plus 50 ng/ml IL13, 100 ng/ml M-CSF, and 100 ng/ml GM-CSF. *n* = 5. Data are representative of two independent experiments **(F–H,J,K)**. NC, no treatment control; *p < 0.05; **p < 0.01; ****p < 0.0001 *via* two-way ANOVA **(B,F,K)** or unpaired *t*-test **(D,H,J)**.

Since NHRI-8-B4 cells showed no advantage in *in vitro* growth but stronger cancer stemness compared to NHRI-1-E4 cells, we assessed the *in vivo* growth capacity of both cell lines in comparison to Hepa1-6. Equal numbers of NHRI-1-E4 or NHRI-8-B4 cells and Hepa1-6 were subcutaneously injected into the left groin and the right groin, respectively, of C57BL/6j mice (Figure 5A). NHRI-8-B4 cells and NHRI-1-E4 gradually grew and Hepa1-6 grew significantly slower than NHRI-8-B4 and NHRI-1-E4 in the same mice (Figure 5B). At the end point, Hepa1-6 tumors were much smaller than NHRI-1-E4 or NHRI-8-B4 tumors (Figures 5C,D). We also inoculated Hepa1-6 alone in C57BL/6j mice to exclude the possible interference in its tumor growth from the other HCC tumors and observed that only 3 out of the 8 inoculated mice developed visible Hepa1-6 tumors within 50 days (Supplementary Figure S6A). In another set of experiment, we inoculated equal numbers of NHRI-1-E4 and NHRI-8-B4 into the left and the right groins, respectively. NHRI-8-B4 cells showed a continuous increase in total flux from day 1 to the end point, whereas NHRI-1-E4 cells showed a downtrend of total flux from day 1 to day 4, followed by an upward trend until the end point (Supplementary Figure S6B). At the end point, NHRI-8-B4 tumors were larger than NHRI-1-E4 tumors (Supplementary Figures S6C,D), which indicated more aggressive *in vivo* growth of NHRI-8-B4-derived tumors compared to those developing from NHRI-1-E4 cells. Progression of orthotopic tumors derived from NHRI-1-E4 and NHRI-8-B4 cells was similar in that NHRI-8-B4 tumors grew faster than NHRI-1-E4 tumors (Figures 5E,F), leading to giant HCC-bearing liver tissue in mice injected with NHRI-8-B4 cells (Figures 5G,H). NHRI-1-E4 tumors developed at lower incidence in the liver at 62.5% (5/8), compared to 100% (5/5) incidence of NHRI-8-B4 tumors after orthotopic inoculation with 1 × 10^5^ tumor cells (data not shown). Histological examination of the subcutaneous tumors showed that NHRI-1-E4 and NHRI-8-B4 tumor cells were similar to Hepa1-6 tumors. The tumors were composed of hyperchromatic cancer cells with high nuclear/cytoplasmic ratio and arranged in thin trabecular patterns. Immunohistochemical stains for the 3 above mentioned subcutaneous tumors were strongly positive for CK18 and albumin in the cytoplasm diffusely, while stains for CK19 in Hepa1-6 were completely negative, and positive in NHRI-1-E4 and NHRI-8-B4 only focally (Supplementary Figure S6E). The histological results echoed the transcriptomic analysis and suggest that NHRI-1-E4 and NHRI-8-B4 harbor features of hepatocyte-originated carcinoma with poor differentiation.

### Cytokine Receptor Expression in NHRI-8-B4 Cells Is Correlated with Aggressive Growth

Analysis of the transcriptome demonstrated that, in comparison to NHRI-1-E4 cells, NHRI-8-B4 cells had upregulated gene expression of numerous cytokine receptors, with especially high levels of interleukin-2 receptor subunit gamma (Il2rg; Figures 5I,J). Il2rg is the common subunit of cytokine receptors for IL-2, IL-4, IL-7, IL-9, IL-15, and IL-21 (Rochman et al., 2009). We therefore examined whether NHRI-8-B4 cells utilized cytokines as growth factors more efficiently than NHRI-1-E4 cells. NHRI-1-E4 and NHRI-8-B4 cells were treated with individual cytokines, including IL-2, IL-4, IL-7, IL-12, IL-13, IL-15, IL-21, M-CSF, GM-CSF, and IL-4 combined with IL-13, for 72 h. Among these cytokines, only IL-4, IL-13, and IL-4 combined with IL-13 dramatically promoted the *in vitro* growth of NHRI-8-B4 cells, but the effect on growth was not observed in NHRI-1-E4 cells (Figure 5K). Expression of *Il4ra*, *Il13ra1*, and *Il13ra2* (data not shown due to no expression in NHRI-1-E4) genes were higher in NHRI-8-B4 cells than in NHRI-1-E4 cells (Figures 5I,J). These results suggest that IL-4 and IL-13 signaling may have roles in NHRI-8-B4 tumor progression.

Besides *Il2rg* and *Il4ra*, *Osmr* (Oncostatin M receptor), *Ghr* (growth hormone receptor), *Tnfrsf22*, and *Tnfrsf9* were upregulated in NHRI-8-B4 cells compared to NHRI-1-E4 cells (Figure 5J). *Osmr* and *Ghr* are known to promote cancer progression (Yu et al., 2019; Strous et al., 2020), and Tnfrsf22 (DcR2), a member of the TNF receptor superfamily, has been shown to be a receptor for TRAIL, but lacks a functional death domain (Sag et al., 2019). Upregulation of *Tnfrsf22* in NHRI-8-B4 cells may result in TRAIL-resistance. *Tnfrsf9* (also known as 4-1BB or *Cd137*) expression has been well-studied in immune cells, but little is known about its function in tumor cells (Glorieux and Huang, 2019). The upregulation of expression of these cytokine receptor genes, as well as surface receptors, may contribute to the rapid progression of NHRI-8-B4 cell derived tumors. Further investigation is needed to characterize this process.

### NHRI-8-B4 Cells Recruit Myeloid Cells to the TME Through Chemokine Expression

**FIGURE 6:**
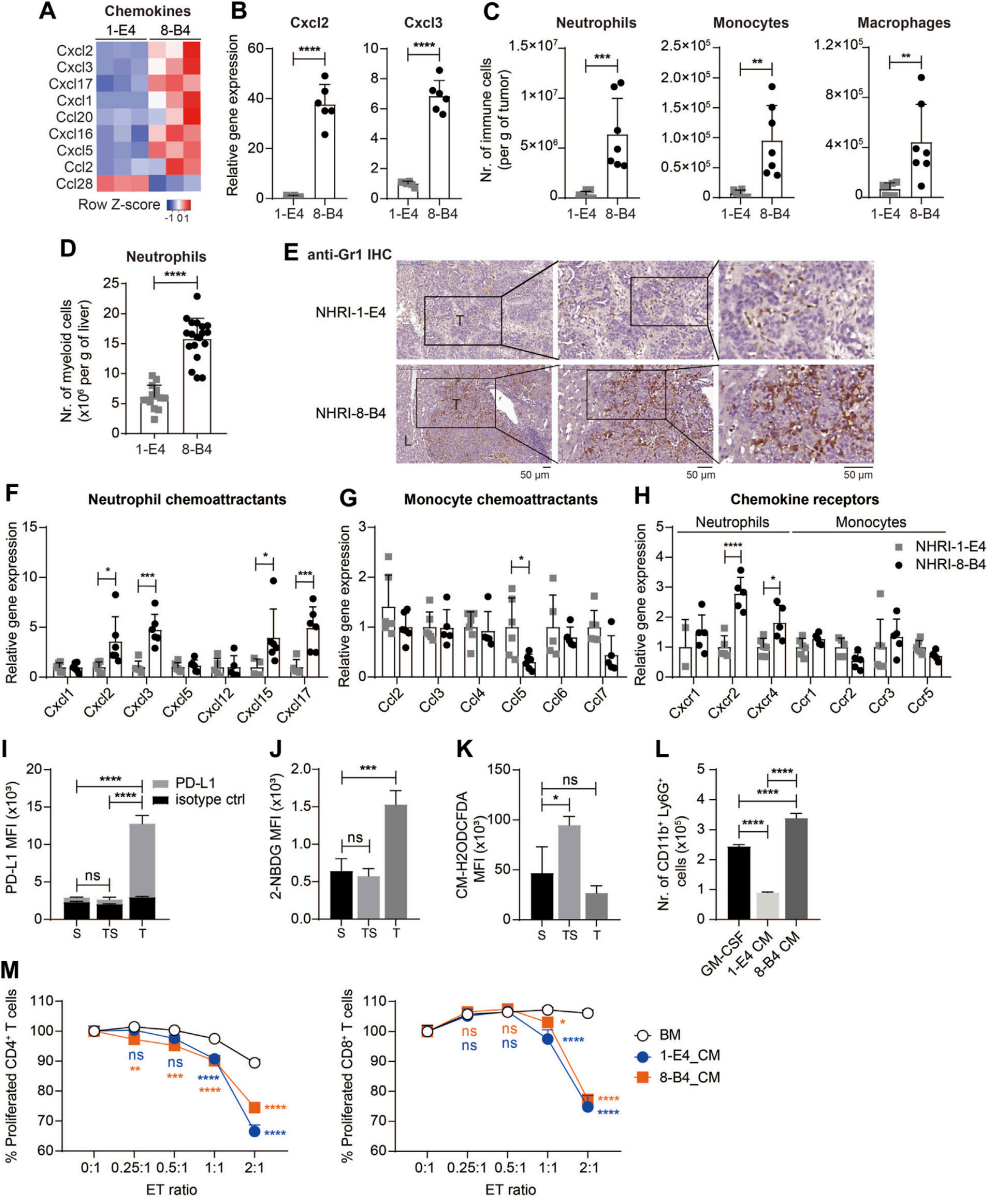
NHRI-8-B4 tumors recruit more neutrophils than NHRI-1-E4 tumors. **(A)** Heatmap of chemokines from RNA-Seq results. **(B)** Levels of *Cxcl2* and *Cxcl3* mRNA in NHRI-1-E4 and NHRI-8-B4 cells. *n* = 6. **(C)** Absolute numbers of neutrophils, monocytes, and macrophages per gram of subcutaneous tumors. *n* = 7. **(D)** Absolute numbers of neutrophils per gram of livers. *n* = 14 for 1-E4 tumors; *n* = 19 for 8-B4 tumors. **(E)** Representative images of intrahepatic tumors with immunostaining for Gr1. All scale bar, 50 μm. T: tumor; L: liver. Levels of chemokine **(F,G)** and chemokine receptor **(H)** mRNA in subcutaneous tissues. *n* = 5. **(I)** PD-L1 expression, **(J)** glucose uptake, and **(K)** ROS in Ly6G^+^CD11b^+^ myeloid cells from subcutaneous NHRI-8-B4 tumors (T) and spleen from tumor-bearing (TS) or normal (S) mice, as detected by PD-L1 antibodies **(I)**, 2-NBDG **(J)**, and CM-H_2_ODCFDA **(K)**, respectively. *n* = 3. **(L)** The number of CD11b^+^ Ly6G^+^ cells present among 1 × 10^6^ bone marrow (BM) cells after a 5 day-culture in GM-CSF (20 ng/ml in RPMI-based medium) or tumor cell-conditioned medium (CM). *n* = 3. **(M)** Proliferation of CD4^+^ and CD8^+^ T cells detected by CFSE staining (2.5 μM for 10 min). *n* = 3. ET (effector to target) ratio indicates the ratio of APC-Ly6G selected bone marrow-derived cells to splenocytes in coculture over 66 h. Data are representative of two independent experiments. ns, not significant; *p < 0.05; **p < 0.01; ***p < 0.001; ****p < 0.0001 *via* unpaired *t*-test **(B–D)** or two-way ANOVA **(F–M)**.

The RNA-seq results revealed that the mRNA levels of several chemokines were more abundant in NHRI-8-B4 cells than in NHRI-1-E4 cells (Figure 6A), especially *Cxcl2* and *Cxcl3*, which were further confirmed by qPCR (Figure 6B). *Cxcl2* and *Cxcl3* are the major chemokines responsible for neutrophil recruitment. Myeloid cells, including tumor-associated macrophages (TAMs), monocytes, and neutrophils, are abundant in the HCC microenvironment and play a decisive role in HCC progression (Wan et al., 2015). We therefore analyzed the recruitment of myeloid cells in the TME in subcutaneous NHRI-1-E4 and NHRI-8-B4 tumors. NHRI-1-E4 and NHRI-8-B4 tumors were grown in the left and the right groins, respectively, of the same mice; therefore, the absolute number of tumor-associated myeloid cells directly reflects the chemoattractant ability of these two tumors. Tumor-associated neutrophils, monocytes, and macrophages were defined as Ly6C^int^ Ly6G^+^ MHC II^−^, Ly6C^high^ Ly6G^−^ MHC II^−^, and F4/80^+^ MHC II^+^, respectively, among CD45^+^ CD11b^+^ cells (Supplementary Figure S7A). We found that more neutrophils, monocytes, and macrophages accumulated in the NHRI-8-B4 TME than in the NHRI-1-E4 TME (Figure 6C). Similar results were observed in the orthotopic model, where more neutrophils, monocytes, and monocyte-derived macrophages (MoMs) were recruited to the NHRI-8-B4 TME than to the NHRI-1-E4 TME (Supplementary Figures S7B,C; Figure 6D). Accumulated neutrophils or monocytes, stained with anti-Gr-1, were far more apparent in the NHRI-8-B4 TME than in the NHRI-1-E4 TME in the liver (Figure 6E). We further analyzed the mRNA levels of chemokines and chemokine receptors in the subcutaneous and intrahepatic tumor tissues. As expected, expression of *Cxcl2*, *Cxcl3*, and corresponding receptors *Cxcr2* and *Cxcr4*, was significantly higher in the NHRI-8-B4 TME than in the NHRI-1-E4 TME (Figures 6F–H, Supplementary Figures S7E–G). In addition, *Cxcl15* and *Cxcl17* levels were enriched in the NHRI-8-B4 tumor tissues (Figure 6F; Supplementary Figure S7E) as well as in NHRI-8-B4 cells (Supplementary Figure S7D). The function of *Cxcl15* in the mouse is unknown, and *Cxcl17* is a chemoattractant factor for macrophages and dendritic cells (Sokol and Luster, 2015), which may explain the accumulation of macrophages in the NHRI-8-B4 TME.

We then analyzed the neutrophils (CD45^+^ CD11b^+^ Ly6G^+^ MHC II^−^) from subcutaneous NHRI-8-B4 tumors and found that tumor-associated neutrophils (T) expressed higher PD-L1 than splenic neutrophils from tumor-bearing mice (TS) or normal mice (Figure 6I). Tumor-associated macrophages also expressed higher PD-L1 and PD-L2 than splenic macrophages (Supplementary Figures S7H,I). Granulocytic myeloid-derived suppressor cells (gMDSCs) in tumor-bearing mice exhibited a higher glycolytic rate and lower levels of reactive oxygen species (ROS) than neutrophils from normal mice did (Jian et al., 2017). We also observed similar phenotypes in NHRI-8-B4 tumor-associated Ly6G^+^ myeloid cells, as demonstrated by results from the 2-NBDG uptake assay and CM-H2DCFDA staining for detection of ROS (Figures 6J,K). There were more CD11b^+^Ly6G^+^ cells recovered from *in vitro* culture of primary bone marrow (BM) cells grown in conditional medium from NHRI-8-B4 cells than from those grown in medium from NHRI-1-E4 cells (Figure 6L). The BM cells cultured in conditional medium from NHRI-1-E4 or NHRI-8-B4 cells exhibited stronger *in vitro* T-cell suppression in comparison to control BM cells (Figure 6M), suggesting that, in addition to showing higher chemoattractant capability in recruitment of neutrophils to the TME compared to NHRI-1-E4 cells, NHRI-8-B4 tumor cells are also able to induce more gMDSC differentiation as potential support for tumor progression.

## Discussion

In this study, we isolated and characterized two phenotypically distinct murine HCC clones derived from liver cancers after SB transposon-mediated integration of *AKT* and *NRAS* oncogenes. The two HCC cell lines expressed higher levels of stemness markers, CD44 and CD133, and also CK18 and CK19 in comparison to the widely used murine HCC cell line, Hepa1-6. CK8 and CK18 are markers for hepatocyte-origin cells, whereas CK7 and CK19 are biliary markers and also are expressed in bipotential hepatic progenitor cells (Eyken et al., 1987). CK19 is detected in tumor sections in more than 20% of HCC patients (Wu et al., 1996; Zhuo et al., 2020). CK19-positive HCC has been shown to have a high metastatic potential, which is associated with a poor prognosis (Uenishi et al., 2003; Zhuo et al., 2020). CK19 expression in HCC can be inherited from the originally transformed hepatic progenitor cells or induced by environmental stimulation e.g. oncogenic growth factors (Yoneda et al., 2011; Rhee et al., 2018). In comparison to Hepa1-6, NHRI-1-E4 and NHRI-8-B4 tumor cells also up-regulated the gene expression of CD56 and epithelial cell adhesion molecule (EpCAM) (data not shown), and therefore we classified them as poorly differentiated HCC type with higher proliferative capability and worse prognosis, which can be a feature of PI3K/AKT-mTOR/RAS-MAPK signaling (Giraud et al., 2021). It has been demonstrated that HCC relies on *de novo* lipogenesis more than cholangiocarcinoma does for tumorigenesis in mouse models (Li et al., 2016). The two novel HCC cell lines harboring progenitor phenotypes and distinct metabolic statuses, can be useful tools for addressing whether lipid metabolism involves in the differentiation of hepatic progenitor cells toward HCC or cholangiocarcinoma or in keeping poorly differentiated status. NHRI-8-B4 showed gene enrichment in β−catenin signaling in comparison to NHRI-1-E4 (Chiang et al., 2008), which may be also a consequence of the aberrant lipid metabolism in NHRI-8-B4 and have further influence on HCC progression (Kim et al., 2015; He and Tang, 2020). The distinct lipid metabolic status between the two cell lines enable to study the influence of lipid in the phenotype and the differentiation of cancer cells and in their involvement in TME.

The SB transposon belongs to Tc1/mariner family and has been shown to arbitrarily insert into palindromic AT-repeats (Vigdal et al., 2002). Besides AT-richness, DNA with bendable structure and deformations of the double helix also determine insertion efficiency of the SB transposon (Vigdal et al., 2002; Geurts et al., 2006). We used Nanopore sequencing to generate long sequencing reads for identification of SB transposon integration sites in the genome of mouse hepatocytes/liver cancer cells. *AKT* and *NRAS* demonstrated higher likelihood for insertion into TA-rich regions. *AKT* insertion into the same TA-rich regions of chromosomes 9, 10, 13, 16, and 18 were found for both HCC clones we developed. NHRI-1-E4 and NHRI-8-B4 also have two identical integration sites for *NRAS* on chromosome 2 and 3. It is likely that the two clones were derived from the same parental progenitor cell before tumorigenesis and therefore had identical integration sites for transgenes. Despite sharing the same genetic composition, the two HCC clones exerted distinct phenotypes regarding fatty acid metabolism and cancer stemness.

NHRI-8-B4 showed up-regulation of the gene expression of fatty acid uptake e.g. SLC27A1 and fatty acid-binding proteins (FABPs). FABPs are important in FA transportation, disposition and metabolism, with some carrying out transcriptional regulation (McKillop et al., 2019). FABP4 is mainly expressed in white and brown adipose tissues, macrophages, and monocytes and upregulation of adipocyte-derived FABP4 promotes tumor progression (Nie et al., 2017; Gharpure et al., 2018). Hepatocytes do not normally express FABP4, but expression occurs in obesity-associated HCC progression and promotes proliferation and migration of human HCC cell lines (Thompson et al., 2018). FABP5, the most widely expressed FABP, is detectable in the epidermis, liver, kidney, lung, adipocytes, brain, and mammary gland (Storch and Corsico, 2008) and is related to tumor progression through activation of peroxisome proliferator-activated receptor signaling (Schug et al., 2007). We observed that levels of FABP4 and FABP5 mRNA were higher in NHRI-8-B4 than in NHRI-1-E4. FABP4 and FABP5 have the capacity to regulate transcription (McKillop et al., 2019) and may regulate tumor progression through both FA metabolism and/or the modulation of gene expression. The roles of FABP4 and FABP5 in HCC progression in NHRI-8-B4 can be explored further via comparison with the phenotypically distinct cell line NHRI-1-E4.

Previous studies have shown that accumulation of LDs is associated with tumor proliferation and aggressiveness, and is a feature of CSCs (Tirinato et al., 2015). Lipid metabolites of stearoyl-CoA-desaturase (SCD) activity are abundant in aggressive HCC samples and could promote tumor invasion and tumor incidence (Budhu et al., 2013), and MUFAs are more abundant in CSCs than in their non-stem counterparts (Li et al., 2017; Mukherjee et al., 2017). In our study, cancer stemness of a cell line correlated with the level of intrinsic accumulation of LDs, which can be enhanced by consumption of exogenous MUFAs. Exogenous MUFAs, but not saturated FAs, promoted tumor proliferation, especially for NHRI-1-E4 (LD^lo^). NHRI-8-B4 (LD^hi^) may generate more MUFAs through higher SCD1 expression and is therefore less dependent on exogenous MUFA consumption for the maintenance of cancer stemness or tumor proliferation. Indeed, NHRI-8-B4 are more susceptible to SCD1 inhibitor treatment than NHRI-1-E4 does regarding cell proliferation and cancer stemness.

NF-κB is the primary pathway regulating the expression and activation of SCD. The expression of SCD also promotes AKT/ERK mediated NF-κB signaling (Fritz et al., 2010; Li et al., 2017). Moreover, the SCD-dependent MUFA level directly regulates CSCs through activation of the Wnt/*β*-catenin pathway (Lai et al., 2017). The NHRI-8-B4 (LD^hi^) characteristics, including upregulation of Scd1 mRNA, activation of the Wnt/β-catenin pathway, and promotion of *β*-catenin downstream target genes, e.g., *Myc* clearly demonstrate that this novel cell line recapitulates the features of CSCs induced by aberrant lipid metabolism.

NHRI-1-E4 harbors more cancer stemness than Hepa1-6, although it shows less cancer stemness than NHRI-8-B4. NHRI-1-E4 upregulates expression of distinct genes, e.g., *Bmi1*, *Sox2*, *Notch1*, compared to NHRI-8-B4, suggesting that NHRI-1-E4 relies on Hedgehog, NF-κB or Notch signaling pathways rather than Wnt/*β*-catenin to maintain its cancer stemness (Yang et al., 2020); these results also imply that the Wnt/*β*-catenin pathway is the major pathway regulating cancer stemness when aberrant lipid metabolism affects CSC differentiation.

IL-4 and IL-13 are Th2 cytokines secreted by macrophages, dendritic cells, mast cells, natural killer T cells, natural killer cells, basophils, eosinophils, and T lymphocytes, and are abundant in the TME. The IL-4Rα/IL-13Rα1 heterodimer (type II IL-4 receptor), predominantly expressed in nonhematopoietic cells, has been identified as the major receptor for both IL-4 and IL-13 in tumors. Type II IL-4 receptor signaling may activate STAT3/6, PI3K/Akt, and ERK1/2 signaling pathways and contribute to tumor progression, and this is the first study linking type II IL-4 receptor signaling with HCC progression. IL-13 can also bind to IL-13Rα2 on tumor cells and enhance tumor invasion and metastasis through activating ERK/activator protein 1 (AP-1) signaling and matrix metalloproteinases (Hallett et al., 2012). Detailed characterization of these paired syngeneic cell lines, NHRI-8-B4 and NHRI-1-E4, reveals the possibility that aberrant lipid metabolism may regulate the expression of chemokines and cytokines, which may further contribute to tumor differentiation, cancer stemness, and immune modulation of in the TME.

Hepa1-6 is the most frequently used syngeneic mouse HCC model, yet Hepa1-6 cells express nearly no MHC class I and growth of Hepa1-6 derived tumors in C57BL/6 mice is not stable, thereby limiting its use as an HCC model for studying immune function or immunotherapy. The two HCC cell lines isolated in this study showed normal expression of MHC class I and stable tumor growth in C57BL/6j mice, making them more suitable for immunological studies than Hepa1-6. The paired HCC cell lines can serve as useful tools for screening therapeutic agents targeting cancer with aberrant lipid metabolism, cancer stemness, and cytokine receptors related to aggressive cancer phenotypes.

## Data Availability

The datasets presented in this study can be found in online repositories. The names of the repository/repositories and accession number(s) can be found below: https://www.ncbi.nlm.nih.gov/, GSE189398.
