# Supplementary material for: Novel Paired Cell Lines for the Study of Lipid Metabolism and Cancer Stemness of Hepatocellular Carcinoma
Source: Front Cell Dev Biol. 2022 May 26;10:821224. doi: 10.3389/fcell.2022.821224 (PMC9204282; doi:10.3389/fcell.2022.821224)
Supplement: Supplementary file 1 [file DataSheet1.docx]

Supplementary Material

# Supplementary materials and methods

**Culture medium**

DMEM-based medium: DMEM medium supplemented with 10% FBS, 1% Penicillin-Streptomycin, 2 mM L-glutamine, 10 mM HEPES, 1 mM sodium pyruvate, and 1% NEAA

RPMI-based medium: RPMI medium supplemented with 10% FBS, 1% Penicillin-Streptomycin, 2 mM L-glutamine, 10 mM HEPES, 1 mM sodium pyruvate, and 50 μM β-mercaptoethanol

RPMI-based medium for fatty acid treatment: RPMI medium supplemented with 1% defatted BSA, 1% Penicillin-Streptomycin, 2 mM L-glutamine, 10 mM HEPES, 1 mM sodium pyruvate, and 50 μM β-mercaptoethanol

**Animal studies and HCC induction by hydrodynamic injection**

Male C57BL/6j mice at 4–5 weeks old were purchased from the National Laboratory Animal Center (Taipei, Taiwan) and were kept in the laboratory animal center (LAC) of NHRI. The two animal facilities are accredited by the Association for Assessment and Accreditation of Laboratory Animal Care (AAALAC) International. C57BL/6j mice were anesthetized with isoflurane mixed with O_2_ before hydrodynamic injection (HDI), and then given HDI of endotoxin-free plasmids dissolved in filtered Dulbecco Phosphate Buffered Saline (DPBS) in a volume equivalent to 8% body weight within 5 seconds. For the mice receiving 2 μg of pCMV(CAT)T7-SB100, 10 μg of pT/Caggs-NRASV12, and 10 μg of pKT2/CLP-AKT-LUC plasmids, the photons emitted from the transduced hepatocytes or tumor cells within the live animals were detected and quantified periodically using IVIS imaging (Caliper Life Sciences, Massachusetts, USA). The mice were injected intraperitoneally with 3 mg of D-luciferin. Ten minutes after injection, the mice were subjected to imaging under anesthesia by isoflurane inhalation. HCC-bearing mice with total flux, measured via IVIS imaging, above 3 × 10^10^ photons/sec were euthanized for liver cell isolation.

**Isolation of HCC clones**

HCC-bearing mice with tumors induced by HDI were anesthetized using isoflurane and perfused with 25 ml of prewarmed Gey’s balanced salt solution (GBSS) via the portal vein at a flow rate of 5 ml/min, with a lamp nearby to maintain the liver temperature at 37 °C. Mice were perfused with 35 ml of DMEM-based medium (10% FBS) with 0.5 mg/ml collagenase IV after the GBSS. Following perfusion, livers were collected and cut to release cells into culture medium. The liver suspension was filtered through a 250 μm cell strainer and then washed in medium three times at 50 g for 1 min. The isolated cells were used to seed two wells of a 6-well plate and then incubated at 37 °C with 5% CO_2_ until colonies formed. Colonies were locally trypsinized with 30-50 μl trypsin and transferred to new wells for continuous growth. The CD45^-^ PDGFR^-^ CD44^+^ cells were further sorted as single cells via FACSorting using Influx (BD Biosciences). Cells grown from a single cell were further characterized using assays for expression of albumin and HCC markers.

**Implantation of HCC cells by subcutaneous injection and intrasplenic injection**

Male C57BL/6j mice at 5-8 weeks old underwent groin implantation of 6x10^5^ HCC cells in 120 μl DPBS containing 16.67% Matrigel via subcutaneous injection or underwent orthotopic implantation of 1x10^5^ HCC cells in 50 μl DPBS via intrasplenic injection. For intrasplenic injection, C57BL/6j mice were anesthetized using isoflurane mixed with O_2_ and subcutaneous injection of buprenorphine for analgesia. The operation was performed under sterile conditions. The spleen was double knotted in the center and cut into two fragments to ensure half of the spleen remained after surgery. Tumor progression was monitored using IVIS imaging twice per week. Mice with up to two subcutaneous tumors with total diameter above 2 cm, or with a liver tumor with the total flux from IVIS imaging above 3 × 10^10^ photons/sec, were euthanized to prevent discomfort associated with large tumors. To analyze orthotopic tumor-associated myeloid cells, 4-8 x10^5^ NHRI-1-E4 cells were injected to induce HCC of similar total liver weight as that induced by 1 x10^5^ NHRI-8-B4 cells.

**Cell isolation and flow cytometry**

Subcutaneous tumors and livers were perfused with a 0.5 mg/ml collagenase IV solution, mechanically disrupted, and digested for 30 min at 37 °C at 250 rpm in GBSS with 0.5 mg/ml collagenase IV, then filtered through a 250-μm cell strainer. For subcutaneous tumors, 1 mg/ml DNase I was added to digest the tumor. Cells were then resuspended in 3 mL DPBS and underlaid with 6.8 mL of 80% Percoll and 5 ml of 25% Percoll for gradient centrifugation for 30 min at 1350 xg at 4 °C. After centrifugation, leukocytes were collected from the interface and subjected to flow cytometry.

All staining for analysis by flow cytometry was performed in the presence of 10 μg/ml Fc block (2.4G2) in fluorescence-activated cell sorting buffer (FACS buffer, phosphate-buffered saline / 2% bovine serum albumin / 0.02% NaN_3_). Acquisition and data analysis were conducted on an Attune NxT flow cytometer (Thermo Fisher Scientific) and using FlowJo software (V.10.0.8r1, FlowJo). Antibodies and dye used for flow cytometric analyses are listed in Supplementary Table 2.

The number of cells for specific cell populations in tumors and liver tissue were adjusted to weight and expressed as absolute number/gram. The tumor-associated neutrophils (CD45^+^ CD11b^+^ Ly6G^+^ Ly6C^int^ MHC II^-^), monocytes (CD45^+^ CD11b^+^ Ly6G^-^ Ly6C^hi^ MHC II^-^), and macrophages (CD45^+^CD11b^+^F4/80^+^MHC II^+^), including Kupffer cells (KC; CD45^+^ CD11b^int^ F4/80^+^ MHC II^+^) and monocyte-derived macrophages (MoM; CD45^+^ CD11b^hi^ F4/80^+^ MHC II^+^), from subcutaneous tumors or liver tumors of tumor-bearing mice were gated using the markers indicated.

**Immunohistochemistry**

Paraffin-embedded liver/tumor tissue sections were deparaffinized, rehydrated, subjected to trypsin-digestion for antigen retrieval, and then incubated with an anti-mouse Ly6G/Ly6C (Gr-1), anti-CK18, CK17/19 or albumin antibody. ImmPRESS anti-Rat or anti-Rabbit IgG, DAB Peroxidase Substrate Kit, and Hematoxylin (all from Vector laboratories) were used for detection and visualization. For Oil Red O staining, 7 μm frozen sections of liver/tumor tissue were fixed in 10% formalin at 4 °C for 10 min, incubated with 0.3 % oil red O solution at room temperature for 15 min, and washed with 60% isopropanol to remove excess dye before mounting with glycerol jelly mounting medium. Images were captured using an automatic digital slide scanner, the Pannoramic MIDI with Plan-Apochromat 20x/0.8 objective (3D HISTECH), in the Pathology Core Laboratory of NHRI and evaluated by an experienced pathologist.

**General ROS detection**

Detection of ROS was performed using 5 μM CM-H_2_DCFDA followed by surface marker staining and flow cytometric analysis.

**Detection of glucose uptake**

Cultured cells were starved in glucose-free RPMI medium at 37 °C with 5% CO_2_ for 1 h, followed by a 1 h treatment with 200 μM 2-NBDG. Glucose uptake was analyzed by flow cytometric analysis after surface marker staining.

***In vitro* T cell suppression assay**

Splenocytes were stained with 2.5 μM Carboxyfluorescein diacetate succinimidyl ester (CFDA-SE) at 37 °C for 10 min at a cell concentration of 1x10^6^/ml in DPBS, followed by a 5 min recovery in RPMI-based medium in 5-fold volume. A total of 1x10^5^ CFSE-labeled splenocytes stimulated by 1 μg/ml anti-CD3/anti-CD28 antibodies were cocultured with various numbers of bone marrow-differentiated Ly6G^+^ myeloid cells for 66 h. The proliferation of T cells was measured by the decrease in the CFSE^+^ population compared to non-stimulated splenocytes.

# Supplementary figures

##
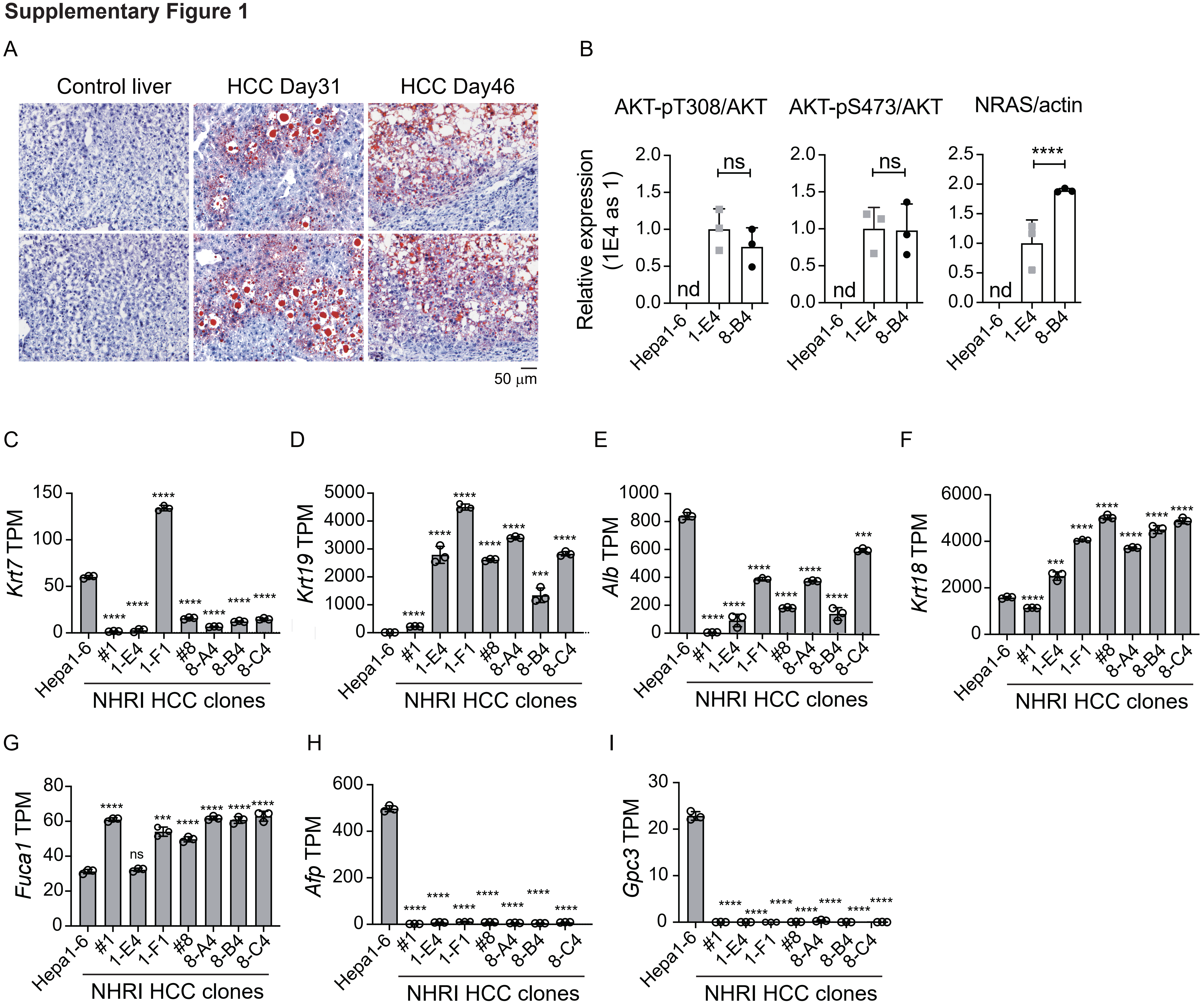


**Supplementary Figure S1. Characterization of HCC clones.** **(A)** Lipid droplets stained by Oil red O in control (normal) liver and *AKT*/*NRAS*-induced HCC bearing liver. **(B)** Quantification of indicated protein expression from Western blot (Figure 1D). Relative protein level compared to that in NHRI-1-E4 cells. nd, not detected; ns, not significant; ****p<0.0001 via one-way ANOVA. Data are representative of three independent experiments. **(C-I)** TPM of Krt7, Krt19, Alb, Krt18, Fuca1, Afp and Gpc3 in Hepa1-6 and several NHRI HCC clones by RNA-seq. n=3. nd, not detected. ns, not significant; *p<0.05; **p<0.01; ***p<0.001;****p<0.0001 via two-way ANOVA compared to Hepa1-6. HCC: hepatocellular carcinoma.





**Supplementary Figure S2. Whole-genome sequencing of NHRI-1-E4 and NHRI-8-B4 cells. (A)** Targeted PCR amplification of Hepa1-6 and several NHRI HCC single clones. NTC: non-template control. **(B)** Sanger sequencing results of fragments amplified from NHRI-1-E4 or NHRI-8-B4 gDNA. Primer sequences for targeted PCR amplification and sanger sequencing are listed in Supplementary Table 4.


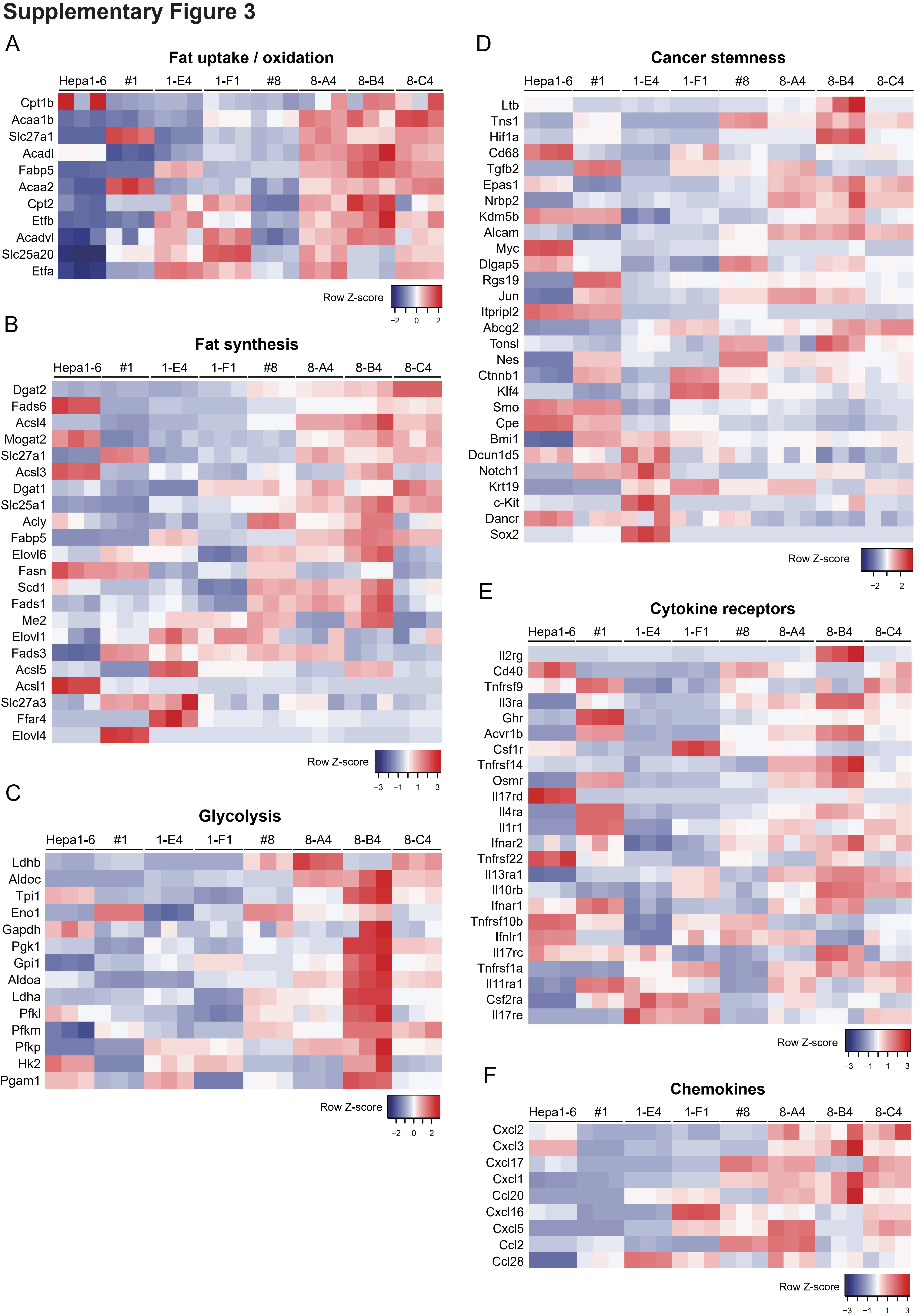


**Supplementary Figure S3. Heatmap of genes involved in (A) fat uptake/oxidation, (B) fat synthesis, (C) glycolysis, (D) cancer stemness, (E) cytokine receptors and (F) chemokines in Hepa1-6 and several NHRI HCC clones from RNA-Seq results.**


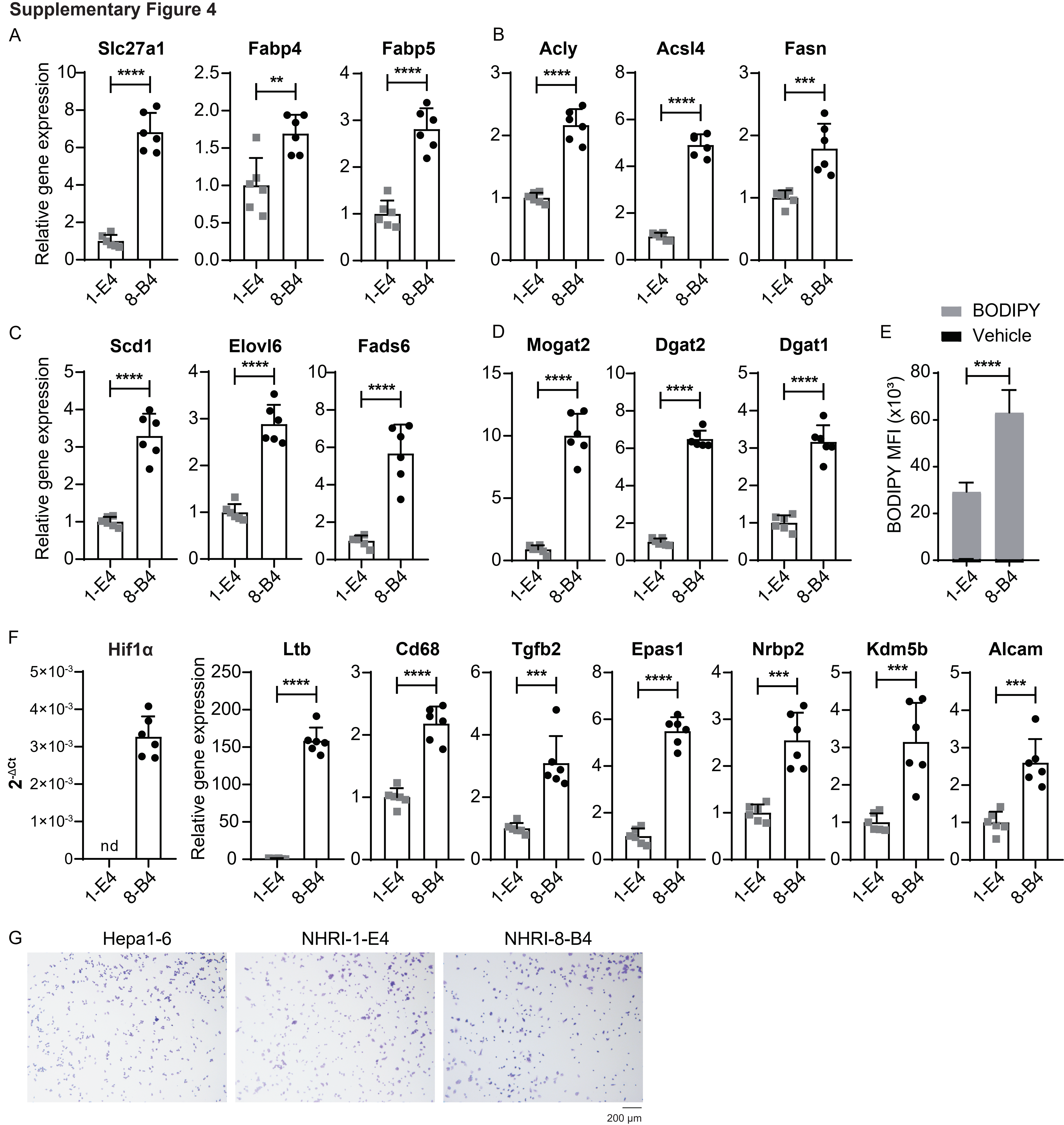


**Supplementary Figure S4. Genes upregulated in NHRI-8-B4 cells compared to NHRI-1-E4 cells.** **(A-D, F)** Quantified levels of mRNA of genes involved in fat uptake **(A)**, synthesis **(B)**, metabolism **(C)** and storage **(D)** and cancer stemness (**F**) in both cell lines. n=6. **(E)** Lipid content in cell lines via BODIPY 493/503 staining. Vehicle: DMSO for BODIPY staining control, indicating low background. n=3. Data are representative of two independent experiments. nd, not detected; **p<0.01; ***p<0.001; ****p<0.0001 via unpaired *t*-test. **(G)**  Crystal violet staining of indicated cells, which originated from the same cell suspension used for Figure 3I.
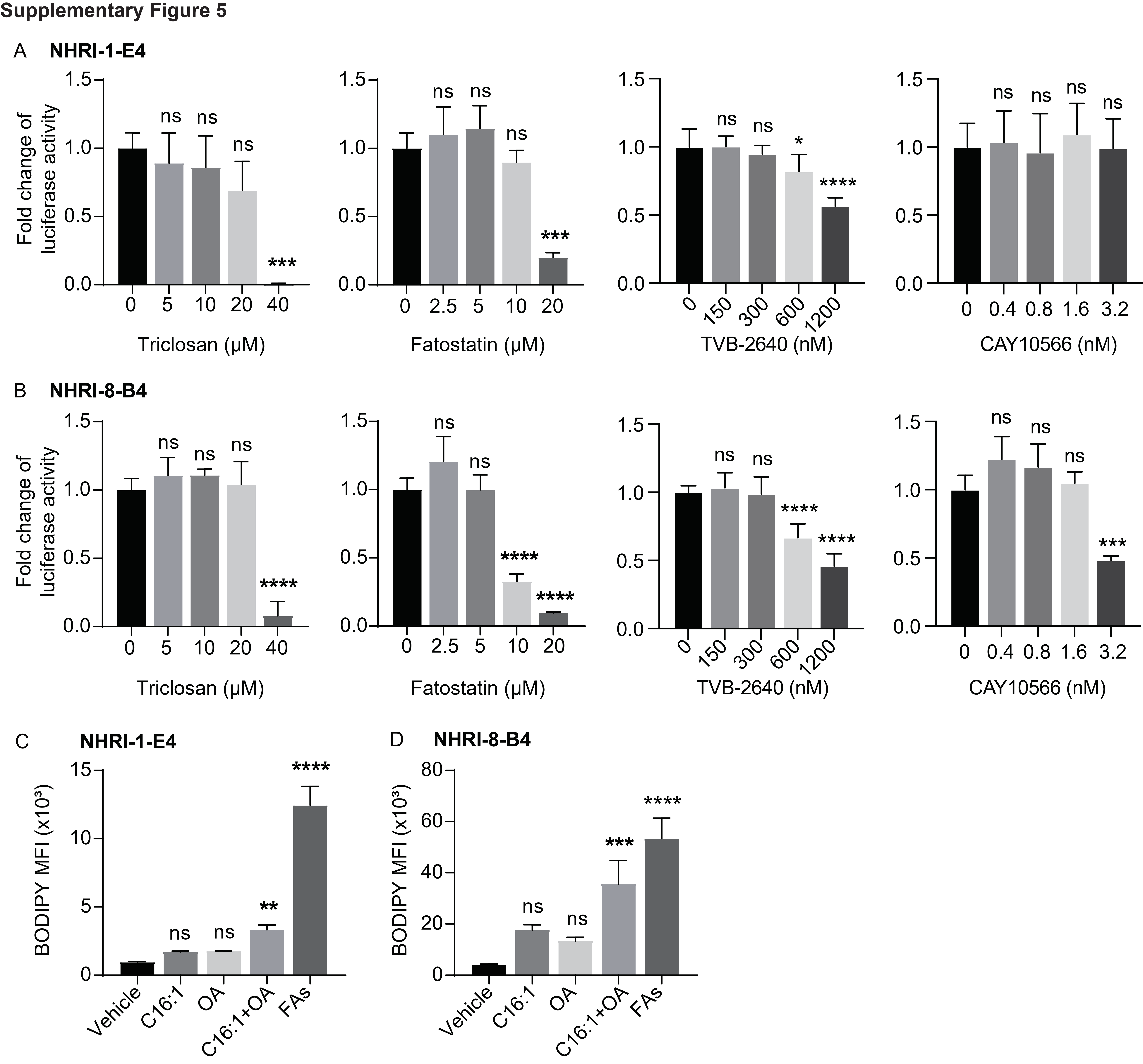


**Supplementary Figure S5. Effects of FASN, SREBP and SCD1 inhibitors on proliferation of HCC cells. (A-B)** Luciferase activity after NHRI-1-E4 (A) and NHRI-8-B4 (B) cells were treated with TVB-2640 and triclosan (FASN inhibitors), or fatostatin (SREBP inhibitor), or CAY10566 (SCD1 inhibitor) at indicated concentrations. n=4. Data are representative of three independent experiments. FASN: Fatty acid synthase; SREBP: Sterol regulatory element-binding protein; SCD1: Stearoyl-CoA desaturase-1. **(C-D)** Lipid content in cell lines treated by indicated fatty acids via BODIPY 493/503 staining. Vehicle: DMSO for BODIPY staining control, indicating low background. n=3. Data are representative of two independent experiments. ns, not significant; *p<0.05; **p<0.01; ***p<0.001; ****p<0.0001 via one-way ANOVA.


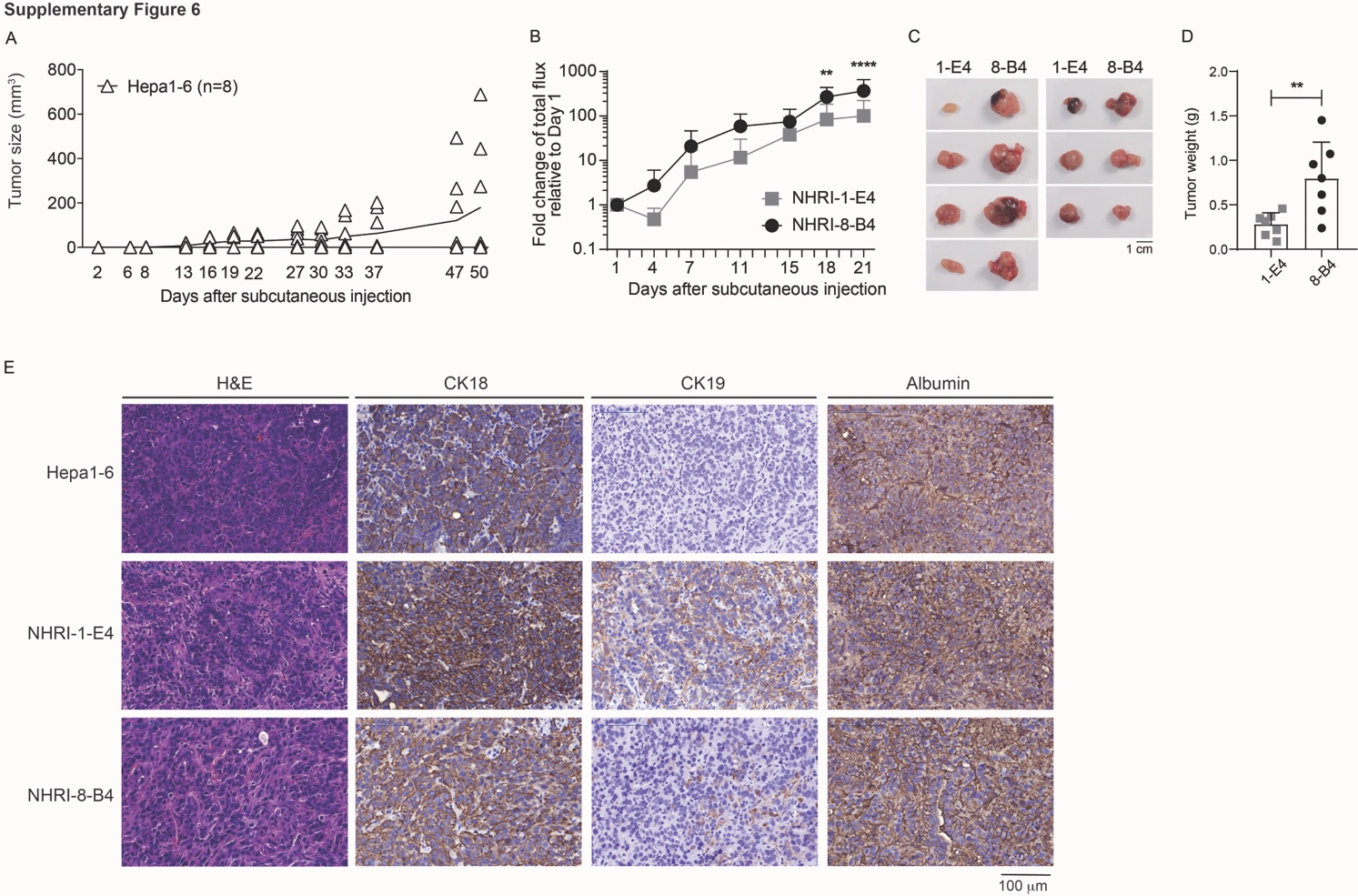


**Supplementary Figure S6. Tumor growth of Hepa1-6, NHRI-8-B4 cells and NHRI-1-E4 cells *in vivo*. (A)** Tumor size of subcutaneously inoculated Hepa1-6 (6x10^5^) cells in groins of C57BL/6j mice (n=8) at indicated time points. **(B)** Tumor growth curves of subcutaneously inoculated NHRI-1-E4 (6x10^5^) and NHRI-8-B4 (6x10^5^) cells in groins of C57BL/6j mice, which were determined by fold change of total flux relative to day 1. **(C-D)** Gross view (C) and tumor weight (g) (D) at day 22 (n=7). **(E)** Representative images of indicated subcutaneous tumors from Figure 5C with H&E staining and immunostaining for CK18, CK19 and albumin.


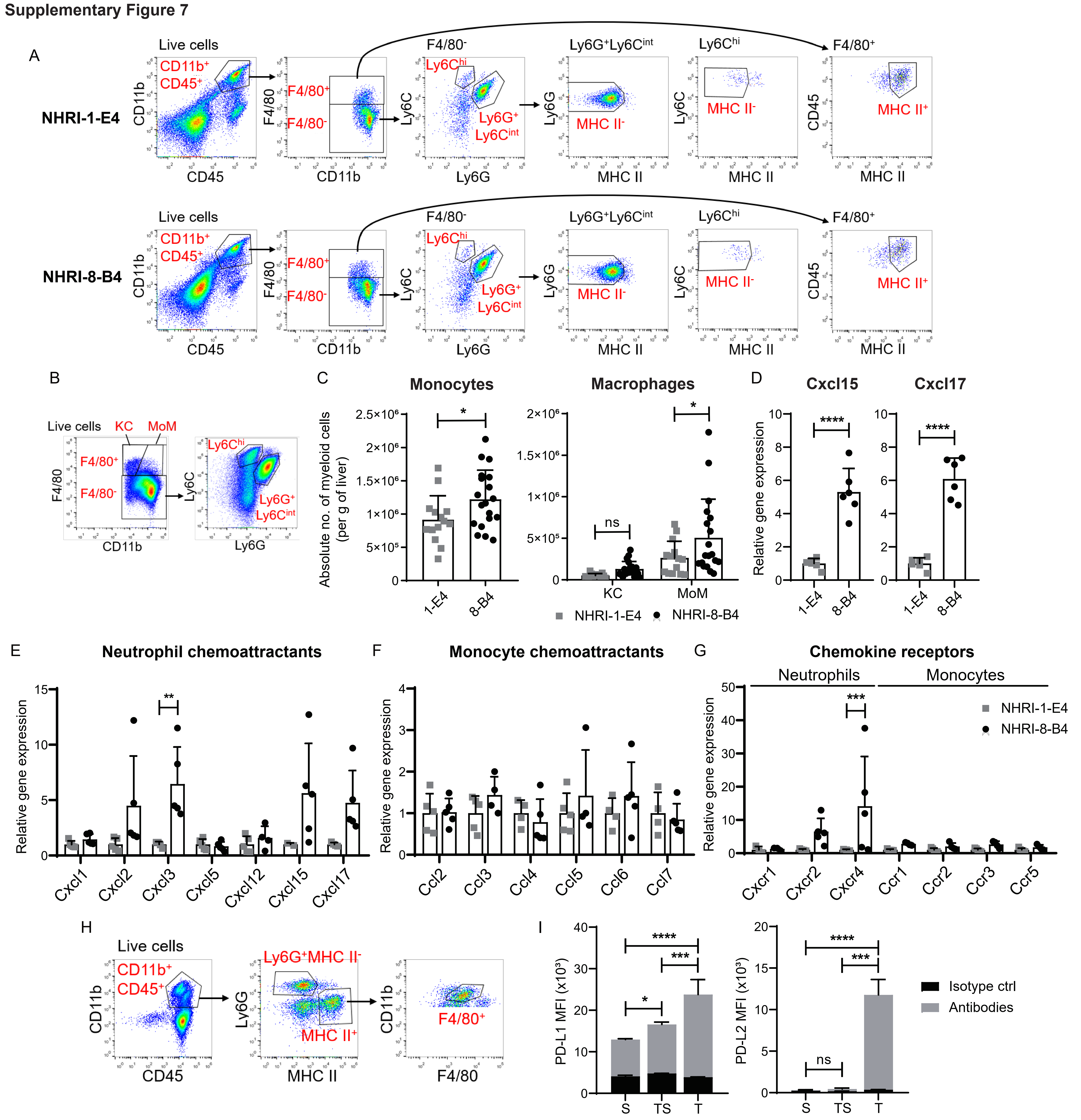


**Supplementary Figure S7. Composition of myeloid cells and chemoattractants in an orthotopic tumor model. (A)** Gating strategy for separation of myeloid cells, CD45^+^ CD11b^+^ Ly6G^+^ Ly6C^int^ MHC II^-^ neutrophils, CD45^+^ CD11b^+^ Ly6C^hi^ MHC II^-^ monocytes, and CD45^+^ CD11b^+^ F4/80^+^ MHC II^+^ macrophages from subcutaneous tumors. **(B)** Gating strategy for separation of CD45^+^ CD11b^low^ F4/80^+^ MHC II^+^ Kupffer cells (KC), CD45^+^ CD11b^hi^ F4/80^+^ MHC II^+^ monocyte-derived macrophages (MoM), CD45^+^ CD11b^+^ Ly6G^+^ Ly6C^int^ MHC II^-^ neutrophils, and CD45^+^ CD11b^+^ Ly6C^hi^ MHC II^-^ monocytes from orthotopic tumors. **(C)** Absolute number of myeloid cells per gram of tumor-bearing liver. n=14 for NHRI-1-E4 tumors; n=19 for NHRI-8-B4 tumors. In order to achieve similar tumor size for comparison of myeloid cell infiltration at the endpoint, we inoculated C57BL/6j mice with 4-8x10^5^ NHRI-1-E4 cells and C57BL/6j mice with 1x10^5^ NHRI-8-B4 tumor cells via intrasplenic injection. The tumor in the liver was isolated when total flux was higher than 1x10^9^ (p/s) and analyzed as the subcutaneous tumor. Neutrophils and monocytes were defined as previously described. Hepatic macrophages were divided into Kupffer cells (KCs) and monocyte-derived macrophages (MoMs) based on CD11b^int^ and CD11b^high^, respectively. ns, not significant; *p<0.05; ****p<0.0001 via unpaired *t*-test for monocytes or two-way ANOVA for macrophages. **(D)** Levels of *Cxcl15* and *Cxcl17* mRNA in NHRI-1-E4 and NHRI-8-B4 cells. n= 6. ****p<0.0001 via unpaired *t*-test. **(E-G)** mRNA levels of chemokine and chemokine receptor in tissue lysates from orthotopic NHRI-1-E4 or NHRI-8-B4 tumors. n=5~6. **p<0.01; ***p<0.001; ****p<0.0001 via two-way ANOVA. **(H)** Representative gating strategy for separation of CD45^+^ CD11b^+^ Ly6G^+^ MHC II^-^ neutrophils and CD45^+^ CD11b^+^ F4/80^+^ MHC II^+^ macrophages from spleen tissue of mice with subcutaneous NHRI-8-B4 tumors. **(I)** PD-L1 and PD-L2 expression in macrophages from (H). n=3. MFI: fluorescence intensity; S: spleen from normal mouse; TS: spleen from tumor-bearing mouse; T: subcutaneous tumor. ns, not significant; *p<0.05; ***p<0.001; ****p<0.0001 via two-way ANOVA. Data are representative of two independent experiments.

**Supplementary Table S1.** Primer sequences for quantitative polymerase chain reaction (qPCR)

| **Target** | **Primer** | **Sequence 5'→3'** |
| --- | --- | --- |
| Actb | Fw | TATAAAACCCGGCGGCGCA |
|  | Rv | TCATCCATGGCGAACTGGTGG |
| Albumin | Fw | CACTCTGGAAAAGTGCTGCG |
|  | Rv | AGGTGCTTTCTGGGTGTAGC |
| Afp | Fw | TGAGGGAATGGCCGACATTTT |
|  | Rv | TCCTCAGAGAATGGGGGAGG |
| Gpc3 | Fw | GGAGCAAGACGTGACCTGAA |
|  | Rv | GCATACGGCCACAGTCCTTA |
| Krt18 | Fw | TGCCAGCTCTGGATTGACTG |
|  | Rv | TCTCCTCAATCTGCTGAGACCA |
| Fuca1 | Fw | GAGGGTGCAGTCGGAAAAGA |
|  | Rv | GCATTGTTATCTTTGTGGCCGA |
| Fabp5 | Fw | CATGGCCAAGCCAGACTGTA |
|  | Rv | GTGCAGACCGTCTCAGTTTTT |
| Fabp4 | Fw | TTTGGTCACCATCCGGTCAG |
|  | Rv | CCAGCTTGTCACCATCTCGT |
| Fabp12 | Fw | GAAGCAGGAGACACTGGGAC |
|  | Rv | TCCTGCTGGCTCTTCCTACT |
| Impa1 | Fw | CGTGCATCGGTTTCCCTTTG |
|  | Rv | CCAACTCGGTCACCAAGAGT |
| Slc27a1 | Fw | TGCCACAGATCGGCGAGTTCTA |
|  | Rv | AGTGGCTCCATCGTGTCCTCAT |
| Acly | Fw | CAGCCAAGGCAATTTCAGAGC |
|  | Rv | CTCGACGTTTGATTAACTGGTCT |
| Acsl4 | Fw | CCTTCCTCTTAAGGCCGGGA |
|  | Rv | TTTGCCATAGCGTTTTTCTTAGATT |
| Fasn | Fw | GCCATTCCAGGTAAATGGGC |
|  | Rv | TGGAGGGTACATCCCAGAGG |
| Scd1 | Fw | GGCGTTCCAGAATGACGTGT |
|  | Rv | CTTTGACAGCCGGGTGTTTG |
| Elovl6 | Fw | GCCCCAAACTTTAGGACGGA |
|  | Rv | CGCTGTCTGCACATTGGATG |
| Fads6 | Fw | CATGATGACCCTGGGGGTTC |
|  | Rv | ACCAAGGGCTTCACCTTCAA |
| Mogat2 | Fw | GGATCCAGAACCGGCTACAG |
|  | Rv | CGATGGGCTTCCCCACTATG |
| Dgat1 | Fw | CCCATACCCGGGACAAAGAC |
|  | Rv | TACTCAGGATCAGCATCACCAC |
| Dgat2 | Fw | GGCTACGTTGGCTGGTAACT |
|  | Rv | TCTTCAGGGTGACTGCGTTC |
| Hif1a | Fw | CCCGTCCACCCATTTCTACC |
|  | Rv | TCGACGTTCAGAACTCATCCTTT |
| Ltb | Fw | ACGGGTCGTTATGGTACACG |
|  | Rv | CCCTCTCCTGTAGTCCACCA |
| Cd68 | Fw | CTTCGGGCCATGTTTCTCTTG |
|  | Rv | AGAGGGGCTGGTAGGTTGAT |
| Tgfb2 | Fw | CCCTCCGAAAATGCCATCCC |
|  | Rv | GGGTTTTGCAAGCGGAAGAC |
| Epas1 | Fw | GTGACCCAAGACGGTGACAT |
|  | Rv | TCCCAAAACCAGAGCCGTTT |
| Nrbp2 | Fw | AAGAAGCGAGGACAAGGCTC |
|  | Rv | TTGTCCTGTCATCCTCGTGC |
| Kdm5b | Fw | AAGAGTTCGCGGACCCCTTC |
|  | Rv | TTTACACGTGTTTGGGCCTCC |
| Alcam | Fw | GCAGTGGGAGCGTCATAAAC |
|  | Rv | TTCTCTGTTTTCATTCGCAGAG |
| Myc | Fw | CAGCGACTCTGAAGAAGAGCA |
|  | Rv | TTGTGCTGGTGAGTGGAGAC |
| Il2rg | Fw | AGCTGAAACGAGAATCCTTCCT |
|  | Rv | CACCACTCCAGGCCGAAAA |
| Il4ra | Fw | ACGTGGTACAACCACTTCCA |
|  | Rv | GAACAGGCAAAACAACGGGA |
| Osmr | Fw | GTCGCCAGGCGGGTAATC |
|  | Rv | CCAAGACTTCGCTTCGGGAT |
| Ghr | Fw | TTACTGAACCCCGGCAGC |
|  | Rv | TCCATACCTGAGACCTCGGA |
| Tnfrsf22 | Fw | ATGGTTCACAGAGATCCCAGA |
|  | Rv | TACGGAAGACAACAACGGACA |
| Tnfrsf9 | Fw | AAGGAAGCAGAACGCTCCTC |
|  | Rv | CTGCAGAAAGTACCAGGCTGA |
| Cxcl1 | Fw | CGGAGAAAGAAGACAGACT |
|  | Rv | AAGCAGAACTGAACTACCA |
| Cxcl2 | Fw | GGGCGGTCAAAAAGTTTGC |
|  | Rv | GTTAGCCTTGCCTTTGTTCAGTATC |
| Cxcl3 | Fw | ACCCAGACAGAAGTCATAGCCA |
|  | Rv | CTTCATCATGGTGAGGGGCT |
| Cxcl5 | Fw | TGCTTAACCGTAACTCCAA |
|  | Rv | ATCCAGACAGACCTCCTT |
| Cxcl12 | Fw | TGCATCAGTGACGGTAAACCA |
|  | Rv | TTCTTCAGCCGTGCAACAATC |
| Cxcl15 | Fw | CTTGGTCTTCCTGCTTGA |
|  | Rv | CGGTGTCCTGATTATCGT |
| Cxcl17 | Fw | AAGAACCAACAGACAGCCACA |
|  | Rv | GCTTGCAGGAACCAATCTTTGC |
| Ccl2 | Fw | AGCCAACTCTCACTGAAG |
|  | Rv | CTCTCCAGCCTACTCATTG |
| Ccl3 | Fw | CAGACACCAGAAGGATACAA |
|  | Rv | TACAGAGAAGAACAGCAAGG |
| Ccl4 | Fw | ACTTCCTGCTGTTTCTCTT |
|  | Rv | ACTGCTGGTCTCATAGTAATC |
| Ccl5 | Fw | CCTGTCATTGCTTGCTCTA |
|  | Rv | ATGCTGATTTCTTGGGTTTG |
| Ccl6 | Fw | TTCGCCCTGCCACAATAGAG |
|  | Rv | ATTTCACCCCAAGAGCCCAG |
| Ccl7 | Fw | GCTGCTTTCAGCATCCAAGTG |
|  | Rv | CCAGGGACACCGACTACTG |
| Cxcr1 | Fw | CCAGCTGGTGCCTCAGATCAA |
|  | Rv | GCCTGCCTGTTGGTTATTGG |
| Cxcr2 | Fw | ATCTTGGTATGCCTACTGATAG |
|  | Rv | TTCTACTTCCTCATTGTCACTT |
| Cxcr4 | Fw | CCATGGAACCGATCAGTGTGA |
|  | Rv | TGCCGACTATGCCAGTCAAG |
| Ccr1 | Fw | TCTTCTATTCTTCCTCCTCTG |
|  | Rv | TTGCTTACTCTGCTCACA |
| Ccr2 | Fw | TGCCATCATAAAGGAGCCAT |
|  | Rv | TTTGTTTTTGCAGATGATTCAA |
| Ccr3 | Fw | CTTATAGCACCACCTCAGT |
|  | Rv | CGCATCACAGTTACAACAT |
| Ccr5 | Fw | CCCCTACAAGAGACTCTGGCTC |
|  | Rv | TTTTGGCAGGGTGCTGACAT |

**Supplementary Table S2.** Antibodies

| **Antibodies** | **Clone** | **Isotype** | **Cat. no** | **Company** |
| --- | --- | --- | --- | --- |
| Anti-Akt (pan) | C67E7 | - | #4691 | Cell Signaling |
| Anti-phospho-Akt (Thr308) | D25E6 | - | #13038 | Cell Signaling |
| Anti-phospho-Akt (Ser473) | D9E | - | #4060 | Cell Signaling |
| Anti-N-Ras | C-20 | - | Sc-519 | Santa Cruz |
| Anti-CK7 | - | - | HPA007272 | Atlas Antibodies |
| Anti-CK18 | N2C2 | - | GTX105624 | GeneTex |
| Anti-CK17/19 | D4G2 | - | #12434 | Cell Signaling |
| Anti-Beta Actin | AC-15 | - | A5441 | Sigma-Aldrich |
| Anti-albumin | - | - | GTX102419 | GeneTex |
| Anti-Gr1 | RB6-8C5 | Rat IgG2b, κ | 108402 | BioLegend |
| PerCP/Cyanine5.5 anti-mouse/human CD44 Antibody | IM7 | Rat IgG2b, κ | 103032 | BioLegend |
| PE anti-mouse CD133 Antibody | 315-2C11 | Rat IgG2a, λ | 141203 | BioLegend |
| BV421 Rat Anti Mouse CD326 | G8.8 | Rat IgG2a, κ | 563214 | BD Biosciences |
| CD45 Monoclonal Antibody, PE-Cyanine 7 | 30-F11 | Rat IgG2b, κ | 25-0451-82 | e-Biosciences |
| PE anti-mouse/human CD11b Antibody | M1/70 | Rat IgG2b, κ | 101208 | BioLegend |
| Brilliant Violet 421™ anti-mouse F4/80 Antibody | BM8 | Rat IgG2a, κ | 123137 | BioLegend |
| Brilliant Violet 605™ anti-mouse I-A/I-E Antibody | M5/114.15.2 | Rat IgG2b, κ | 107639 | BioLegend |
| PerCP/Cyanine5.5 anti-mouse Ly-6G Antibody | 1A8 | Rat IgG2a, κ | 127616 | BioLegend |
| APC anti-mouse Ly-6C Antibody | HK1.4 | Rat IgG2c, κ | 128016 | BioLegend |
| PE anti-mouse CD273 (B7-DC, PD-L2) Antibody | TY25 | Rat IgG2a, κ | 107205 | BioLegend |
| PE/Cyanine7 anti-mouse CD274 (B7-H1, PD-L1) Antibody | 10F.9G2 | Rat IgG2b, κ | 124313 | BioLegend |

**Supplementary Table S3.** Reagents

| **Reagents** | **Catalog number** | **Company** |
| --- | --- | --- |
| Bovine Serum Albumin (BSA), Fraction V, Fatty Acid Free for tissue culture | A-421-50 | Gold Biotechnology |
| TVB-2640 | S9714 | Selleckchem |
| Triclosan | 72779 | Sigma-Aldrich |
| Fatostatin | 13562 | Cayman |
| CAY10566 | 10012562 | Cayman |
| Myristic acid | 70079 | Sigma-Aldrich |
| Palmitic acid | 76119 | Sigma-Aldrich |
| Palmitoleic acid | 76169 | Sigma-Aldrich |
| Oleic acid | O1383 | Sigma-Aldrich |
| Erucic acid | 45629 | Sigma-Aldrich |
| Zombie NIR™ Fixable Viability Kit | 423106 | BioLegend |
| BODIPY 493/503 | D3922 | Thermo Fisher Scientific |
| Collagenase, type IV | C5138 | Sigma-Aldrich |
| D-luciferin Firefly | L-8220 | Biosynth Chemistry & Biology |
| Matrigel | 356237 | Corning |
| Proleukin (aldesleukin, IL-2) | - | Clinigen |
| Recombinant Mouse IL-4 (carrier-free) | 574304 | BioLegend |
| Recombinant Mouse IL-7 (carrier-free) | 577802 | BioLegend |
| Recombinant Mouse IL-12 (p70) (carrier-free) | 577002 | BioLegend |
| Recombinant Mouse IL-13 (carrier-free) | 575902 | BioLegend |
| Recombinant Mouse IL-15 (carrier-free) | 566302 | BioLegend |
| Recombinant Mouse IL-21 (carrier-free) | 574502 | BioLegend |
| Recombinant Murine M-CSF | 315-02 | PEPRO TECH |
| Recombinant Murine GM-CSF (carrier-free) | 576306 | BioLegend |
| 2-NBDG | N13195 | Thermo Fisher Scientific |
| CM-H_2_DCFDA | C6827 | Thermo Fisher Scientific |
| CellTrace CFSE Cell Proliferation Kit | C34554 | Thermo Fisher Scientific |

Fatty acids were prepared at 100 mM in DMSO as stock and at final concentration of 150 μM in culture medium for treatment.

**Supplementary Table S4.** Primer sequences for targeted PCR amplification and sanger sequencing

| Insertion cassette | Junction | PCR primers (5'→3') | Sequencing primer (5'→3') |
| --- | --- | --- | --- |
| Chr2_NRAS | 3’ | cgaaggcttcctctgtgt | gacagggtgttgaagatgc |
|  |  | GACCACTAACAGGAGAATG |  |
| Chr3_NRAS | 5’ | CACGTCTGCACAGTTATG | cgaccatggtaatagcga |
|  |  | cgaccatggtaatagcga |  |
|  | 3’ | gacagggtgttgaagatgc | gacagggtgttgaagatgc |
|  |  | CAGGAGTGGTCTGCAGAG |  |
| Chr9_AKT | 5’ | GAGCAGAGTTGTATCGCA | cctgttgattcaccccta |
|  |  | cctgttgattcaccccta |  |
|  | 3’ | gtgtgtgagaggtcctatg | GGAAAGATCGCCGTGTAA |
|  |  | CCTTCCATAGCAACCTGAC |  |
| Chr10_AKT | 5’ | CTGCACTGTAGCTGGGAG | cctgttgattcaccccta |
|  |  | gaggaggaagtagcgtgg |  |
|  | 3’ | gtgtgtgagaggtcctatg | GGAAAGATCGCCGTGTAA |
|  |  | GCTGCCGTAAATCCTAGTC |  |
| Chr13_AKT | 5’ | GACGCACACGCTTATAGT | cctgttgattcaccccta |
|  |  | gaggaggaagtagcgtgg |  |
|  | 3’ | gtgtgtgagaggtcctatg | GGAAAGATCGCCGTGTAA |
|  |  | CAGAATGCCATACAGAACAA |  |
| Chr16_AKT | 5’ | GCGATCCATTCCATCCAATAGC | cctgttgattcaccccta |
|  |  | cctgttgattcaccccta |  |
|  | 3’ | gacatagcttactgggacg | GGAAAGATCGCCGTGTAA |
|  |  | GATGTTCCAACCAGTAAGAAGG |  |
| Chr18_AKT | 5’ | GCTTTGAGCGCCATAACACA | GCTTTGAGCGCCATAACACA |
|  |  | gaggaggaagtagcgtgg |  |
|  | 3’ | TGACTCCCAAGAGGTACACCA | TGACTCCCAAGAGGTACACCA |
|  |  | gtgtgtgagaggtcctatg |  |
